# Accessory enzymes of hypercellulolytic *Penicillium funiculosum* facilitate complete saccharification of sugarcane bagasse

**DOI:** 10.1186/s13068-021-02020-x

**Published:** 2021-08-26

**Authors:** Olusola A. Ogunyewo, Pooja Upadhyay, Girish H. Rajacharya, Omoaruemike E. Okereke, Laura Faas, Leonardo D. Gómez, Simon J. McQueen-Mason, Syed Shams Yazdani

**Affiliations:** 1 Microbial Engineering Group, International Centre for Genetic Engineering and Biotechnology, New Delhi, 110067 India; 2 DBT-ICGEB Centre for Advanced Bioenergy Research, International Centre for Genetic Engineering and Biotechnology, New Delhi, 110067 India; 3 Biotechnology Advanced Research Centre, Sheda Science and Technology Complex (SHESTCO), Abuja, Nigeria; 4 Department of Biology, Centre for Novel Agricultural Products, CNAP, University of York, York, YO10 5DD UK

**Keywords:** Sugarcane bagasse, Proteomics, Engineered *Pf*Mig1^88^, CAZymes, Biomass saccharification, Quantitative mass spectrometry

## Abstract

**Background:**

Sugarcane bagasse (SCB) is an abundant feedstock for second-generation bioethanol production. This complex biomass requires an array of carbohydrate active enzymes (CAZymes), mostly from filamentous fungi, for its deconstruction to monomeric sugars for the production of value-added fuels and chemicals. In this study, we evaluated the repertoire of proteins in the secretome of a catabolite repressor-deficient strain of *Penicillium funiculosum*, *Pf*Mig1^88^, in response to SCB induction and examined their role in the saccharification of SCB.

**Results:**

A systematic approach was developed for the cultivation of the fungus with the aim of producing and understanding arrays of enzymes tailored for saccharification of SCB. To achieve this, the fungus was grown in media supplemented with different concentrations of pretreated SCB (0–45 g/L). The profile of secreted proteins was characterized by enzyme activity assays and liquid chromatography–tandem mass spectrometry (LC–MS/MS). A total of 280 proteins were identified in the secretome of *Pf*Mig1^88^, 46% of them being clearly identified as CAZymes. Modulation of the cultivation media with SCB up to 15 g/L led to sequential enhancement in the secretion of hemicellulases and cell wall-modifying enzymes, including endo-β-1,3(4)-glucanase (GH16), endo-α-1,3-glucanase (GH71), xylanase (GH30), β-xylosidase (GH5), β-1,3-galactosidase (GH43) and cutinase (CE5). There was ~ 122% and 60% increases in β-xylosidase and cutinase activities, respectively. There was also a 36% increase in activities towards mixed-linked glucans. Induction of these enzymes in the secretome improved the saccharification performance to 98% (~ 20% increase over control), suggesting their synergy with core cellulases in accessing the recalcitrant region of SCB.

**Conclusion:**

Our findings provide an insight into the enzyme system of *Pf*Mig1^88^ for degradation of complex biomass such as SCB and highlight the importance of adding SCB to the culture medium to optimize the secretion of enzymes specific for the saccharification of sugarcane bagasse.

**Supplementary Information:**

The online version contains supplementary material available at 10.1186/s13068-021-02020-x.

## Background

The production of biofuels such as bioethanol from lignocellulosic biomass continues to be of great importance because cellulosic ethanol-based biorefinery has the potential to substitute gasoline, promote rural development, and reduce greenhouse gases [1, 2]. Sugarcane bagasse (SCB), a crushed fibrous residue of sugarcane stalks remaining after juice extraction, is one of the largest cellulosic agro-industrial wastes produced in many countries with a worldwide annual production of approximately 54 million tons [3]. It is a readily available by-product of sugar processing mills whose valorization will have a long-lasting effect on the sustenance of sugar mills, which are struggling to keep their profit margins reasonably high. Bagasse, which has a relatively high carbohydrate content in the form of cellulose and hemicellulose, is a cheap and readily available side product from the sugar manufacture. SCB is a potential feedstock for the production of fuels such as bioethanol and other value-added chemicals (like citric acid, glutamic acid, lactic acid, xylitol, bioplastics and polyhydroxyalkanoates) from the sugars produced by the hydrolysis of its constituent polysaccharides [4, 5].

Lignocellulolytic enzymes have attracted much interest in enzymology for the degradation of lignocellulose into fermentable sugars. They account for a significant percentage of the total costs associated with the bioconversion of lignocellulosic feedstocks into renewable fuels and chemicals [6]. Process cost is generally measured with a top-down approach that accounts for the type of feedstock, enzyme load, and overall biofuel yield in addition to actual enzyme cost [7]. One approach employed in industry to reduce the enzyme cost involves on-site production of enzymes in a unit attached to the biorefinery plant, using carbon catabolite derepressed filamentous fungi to produce enzymes. This integrated enzyme production approach uses incoming lignocellulosic biomass as substrate for on-site enzyme production [8–10].

In previous studies, we have identified *Penicillium funiculosum* NCIM1228 as a host for the production of potent lignocellulolytic enzymes for deconstruction of diverse pretreated cellulosic feedstocks [11, 12]. Its secretome exhibited high saccharification potential when compared with secretomes from other fungal strains like *Trichoderma reesei* RUT C-30. The utilization of lignocellulosic biomass as the carbon source for the induction of lignocellulolytic enzymes in fungal strains should contribute to reduce the cost for production of enzymes. Additionally, the use of biomass to induce enzyme secretion in the fungus will produce a consortium of tailored enzymes specific for saccharification of that particular biomass. However, our previous attempts to utilize pretreated biomass as the sole carbon source for the production of cellulolytic enzymes by *P. funiculosum* (NCIM 1228) were unsuccessful and negligible enzyme activity was obtained [13]. Proteomic analysis of the secretome produced revealed that the secretome contained many stress-related proteins instead of cellulolytic proteins, compared with the secretome produced using crystalline cellulose as the carbon source [13]. Furthermore, the presence of different cellulosic sources revealed the importance of utilizing crystalline substrate as carbon source in the induction media, in order to achieve a highly active cocktail for saccharification of cellulosic feedstocks [13]. Avicel, which is the most crystalline of the evaluated polymeric substrates induced more of core cellulases and swollenin, compared to wheat bran where the most abundantly expressed proteins were majorly categorized as hemicellulases. However, the composite mixture of Avicel and wheat bran produced substantial blend of cellulases and hemicellulases in various proportions with outstanding saccharification potential [13].

The present study achieves a deeper understanding on the use of pretreated SCB as carbon source on the fungal enzyme production machineries and on the efficiency of the enzyme cocktail produced for the saccharification of pretreated biomass. The strain used in this study was the *Mig1* gene deleted variant of *Penicillium funiculosum* NCIM1228, i.e., *Pf*Mig1^88^, where the alleviation of carbon catabolite repression mechanism led to more than twofold increase in protein secretion, enzyme titer and saccharification performance [12, 14, 15]. Here, we present an in-depth profile of the enzymatic and accessory protein repertoire of engineered *Pf*Mig1^88^. We characterize and compare the secretomes of *Pf*Mig1^88^ produced in response to varying concentrations of alkali-pretreated SCB. Using a combination of both, biochemical and proteomic approaches, we performed a detailed characterization of the proteins present in the secretory cocktail of the fungus. We characterized the enzyme activities of the key biomass-degrading enzymes alongside the biomass saccharification efficiencies of the different secretomes, while the relative enzyme abundance was estimated by label-free quantitative proteomics. The results presented here identify the most relevant proteins for the conversion of SCB to fermentable sugars from *Pf*Mig1^88^ and highlight their biotechnological relevance. Most importantly, it was discovered that the presence of these enzymes in the secretome led to almost complete saccharification of pretreated SCB to monomeric sugars, which can subsequently be converted to bioethanol or other value-added chemicals. The dataset reported here could be useful for developing effective technology for utilization of sugarcane bagasse towards second-generation biofuels.

## Results and discussion

### Composition analysis of alkaline-pretreated sugarcane bagasse

**Fig. 1:**
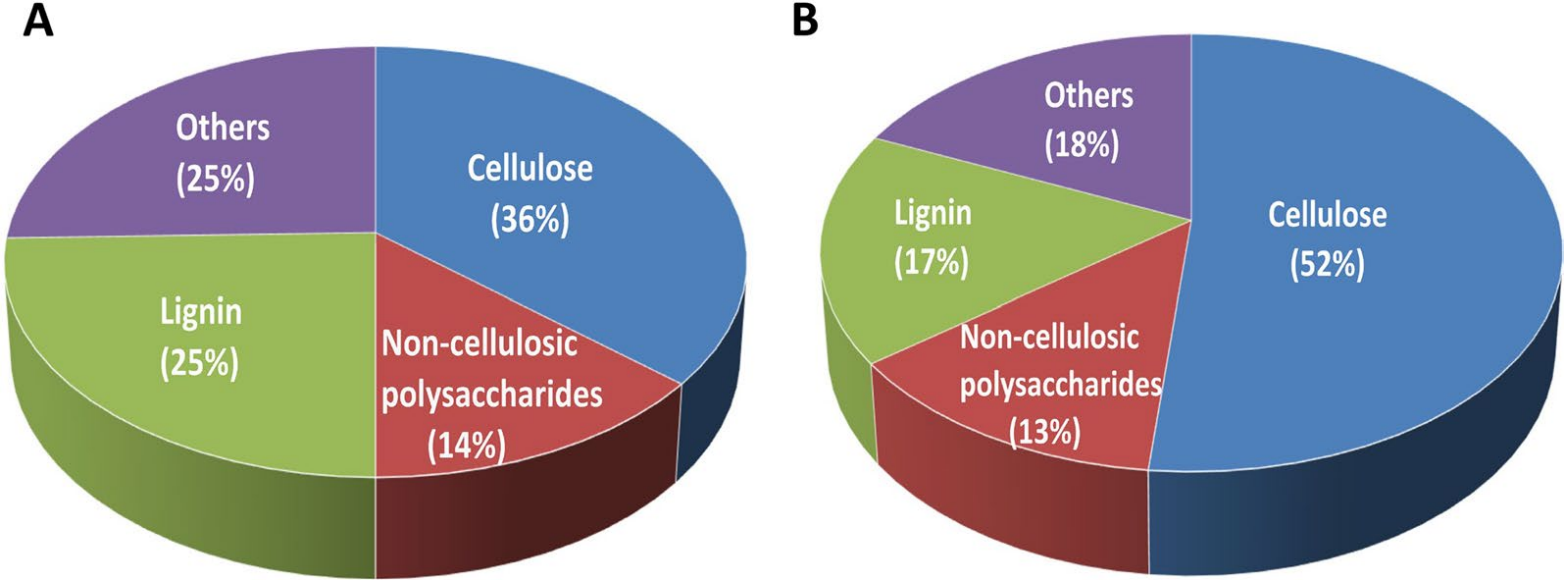
The compositional analysis of the carbohydrate and lignin contents of SCB before pretreatment (**A**) and after pretreatment using 0.4 N NaOH (**B**). The non-cellulosic polysaccharides correspond to the hemicellulose and pectin

The utilization of agricultural cellulosic feedstocks for the production of value-added products involves the biochemical conversion of plant biomass into simple sugars using enzymes. These enzymes, which include cellulases, hemicellulases, and other accessory proteins, are required to degrade the structural polysaccharides into monomeric sugars for microbial fermentation into the desired final products [16]. However, this process is hampered by the recalcitrance conferred by the composition and architecture of the plant cell wall of lignocellulose to enzymatic degradation [17]. Therefore, in order to effectively utilize the SCB as carbon source for fungal growth and production of monomeric sugars, the alkaline pretreatment method was employed to alter the chemical and physical structure of the hemicellulose and lignin matrix, providing a more accessible cellulose component. The compositional analyses of the carbohydrate and lignin contents of SCB before and after pretreatment using 0.4 N NaOH are presented in Fig. 1A, B. The results show that the alkaline pretreatment was effective to reducing the lignin content in the biomass, thereby increasing the cellulose content in the recovered substrate while marginally affecting the total hemicellulose content. We observed an increase in the proportion of cellulose from 36 to 52% and a reduction in lignin content from 25 to 17% in the recovered substrate. Although it was noticed that there was no significant change in the overall hemicellulose content after the alkaline pretreatment, analysis of the monosaccharides from the non-cellulosic polysaccharides including hemicelluloses and pectins showed a change in the sugar profile after pretreatment (Table 1). While minor monosaccharides were reduced, an increase in the concentrations of xylose and glucose, which are the major fractions of the hemicellulose and are useful for microbial growth, was noticed (Table 1). Conversely, the arabinose concentration remained constant. Overall, the pretreatment produced an enrichment in the cellulose fraction relative to all other components in the SCB which makes the partially delignified sugarcane bagasse more susceptible to enzymatic attack during microbial cultivation as well as during saccharification.

**Table 1:** Concentration of non-cellulosic polysaccharides matrix before and after pretreatment (μg monosaccharides/mg biomass)

Component	Untreated	Pretreated
Fucose	0.28 ± 0.06	1.00 ± 0.06
Arabinose	26.96 ± 0.80	25.87 ± 0.61
Rhamnose	1.72 ± 0.28	0.00 ± 0.00
Galactose	10.46 ± 0.81	4.94 ± 0.21
Glucose	24.37 ± 0.66	28.91 ± 0.11
Xylose	66.51 ± 0.31	67.91 ± 1.35
Mannose	1.77 ± 0.28	0.26 ± 0.02
Galacturonic acid	4.37 ± 0.56	0.47 ± 0.25
Glucuronic acid	0.69 ± 0.08	0.39 ± 0.01

### Proteomic profiling of *Pf*Mig1^88^ secretome for sugarcane bagasse deconstruction

**Fig. 2:**
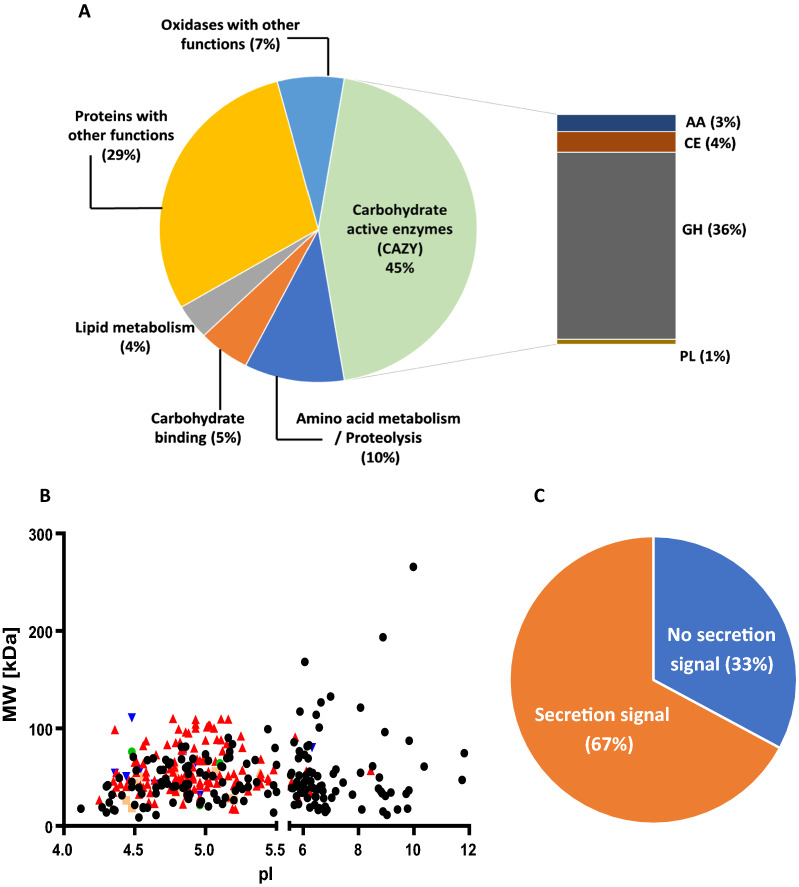
Compositional analysis of total proteins detected in secretome of *Penicillium funiculosum* (*Pf*Mig1^88^). **A** Functional classification of proteins identified in secretome of *Pf*Mig1^88^. **B** A plot of calculated molecular weight against the isoelectric point (pI) of the identified proteins. Proteins annotated with different functions are indicated with different symbols. Data points in inverted blue triangle indicate auxiliary activities-related enzymes (AAs); orange-colored rectangles are carbohydrate esterases (CEs); red colored triangles indicate the glycoside hydrolases (GHs); green colored circles indicate the polysaccharide lyases (PLs). All other proteins that are non CAZYmes are represented as black colored circles. **C** Categorization of proteins according to the presence of signal peptide that led proteins towards the secretory pathway

Since the main goal of this study was to identify and explore the largest possible number of secreted proteins involved in degradation of SCB by the catabolite repressor-deficient fungus *Pf*Mig1^88^, we carried out a comparative proteomic analysis using secretomes of the fungus modulated with different concentrations of pretreated SCB. This was done by comparing the obtained mass spectra against an in-house predicted proteins obtained from the draft genome sequence of the fungus available in our laboratory as reported previously [11]. From the analysis, we were able to confidently identify 280 proteins validated at 1% FDR first in the secretome of *Pf*Mig1^88^ when cultivated in the inducing media containing only microcrystalline cellulose and wheat bran (Additional file 1: Table S1). This condition was subsequently used as control for other experiments. The number of proteins identified were significantly higher than the number reported in our previous work with the native strain of this fungus (*P. funiculosum* NCIM1228), where only 195 proteins were identified [13]. The difference in the number of detected proteins could be attributed to the sensitivity of the mass spectrometer used, as well as higher induction of a larger subset of enzymes during cultivation of the derepressed version of NCIM1228, i.e., *Pf*Mig1^88^. Functions were subsequently assigned to the identified proteins from the genus *Talaromyces* (the closest neighbor to NCIM1228 based on the genome sequence homology) using Blast2go suite (Fig. 2A). The molecular weights of all the identified proteins were in the range of 9–331 kDa while their pIs were in the range of 4.1–11.9 with the carbohydrate active enzymes (CAZymes) mostly in the acidic range of 4–6 (Fig. 2B). Out of the 280 identified proteins, 126 are classified as CAZymes constituting 45% of the total proteins secreted. Accessory proteins involved in carbohydrate binding were 5% of the total secretome, while proteins involved in lipid metabolism and amino acid metabolism were 4% and 10%, respectively. Similarly, 178 out of the 280 identified proteins contained the N-terminal Sec-dependent secretion signal (Fig. 2C). The proteins without signal peptides in the secretome may be extracellular proteins secreted by alternative secretion pathways or intracellular proteins leaked as earlier reported [9, 18].

**Fig. 3:**
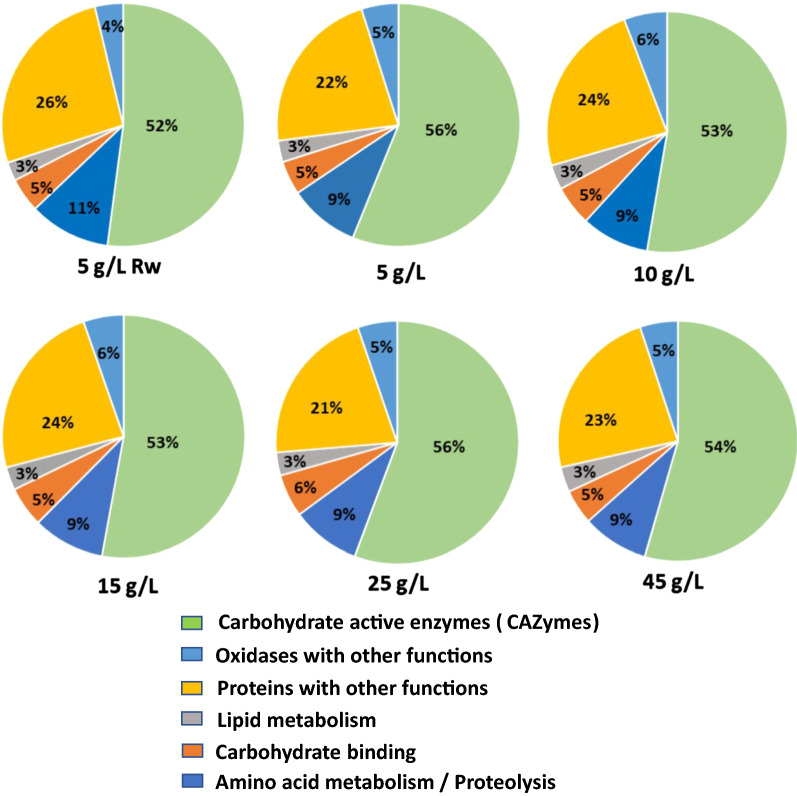
Functional classification of proteins identified in secretome of *Penicillium funiculosum* (*Pf*Mig1^88^) modulated with different concentrations of untreated (Rw) and pretreated sugarcane bagasse

A further analysis of the proteins produced by *Pf*Mig1^88^ with different concentrations of SCB included in the cultivation media showed 6–18% reduction in the number of total proteins identified under each condition compared with the control. A total of 240 proteins were identified in the secretome of *Pf*Mig1^88^ when the production media was supplemented with 5 g/L of raw or pretreated SCB (Additional file 2). Similarly, 241, 261, 228 and 235 proteins were detected in the secretomes containing pretreated SCB at 10, 15, 25 and 45 g/L concentrations, respectively (Additional file 2). Functional annotation of the proteins identified under these conditions showed an increase in the proportion of carbohydrate active enzymes in the secretomes (51–56%) with respect to the control (45%) (Fig. 3). The difference in the proportion of the CAZymes especially in production media supplemented with pretreated SCB could be attributed to increased accessibility of the cellulose and hemicellulose component of the biomass in the cultivation media. The CAZymes cut across the core cellulases such as cellobiohydrolases (GH6-CBM1, GH7-CBM1), endoglucanases (GH5-CBM1, GH7-CBM1), β-glucosidases (GH1, GH3) and remarkably high xylanolytic enzymes distributed across the GH3, GH5, GH11, GH30 and GH43 subfamilies. The higher number of xylan-degrading enzymes detected across the different secretomes was similar to that reported for *Aspergillus niger* cultivated on pretreated SCB. This diversity in xylanases has been attributed to the complexity of the acetylated glucuronoarabinoxylan present in SCB [9, 19]. Furthermore, amidst the CAZymes detected were pectin-degrading enzymes that included rhamnogalacturonase (GH28) and rhamnogalacturonan acetylesterase (CE12), both responsible for degradation of rhamnogalacturonan I, the main pectic component in sugarcane cell walls [20]. β-Galactosidase (GH35) and pectate lyase (PL1) were also present in the proteome. Chitinases (GH18) and β-1,3-glucan-active enzymes (GH16 and GH17), which are required for the organization and remodeling of fungal cell wall polysaccharides, were also detected across all the secretomes. The presence of some β-1,3-glucanases in the secretomes suggests possible activity against mixed β-1,3-1,4-glucan present in sugarcane cell walls [21]. While there were no changes in the proportion of the enzymes involved in carbohydrate binding, amino acid metabolism and lipid metabolism across all the secretomes, we observed a significant reduction in the proportion of proteins involved in other metabolic functions (Fig. 3). These results showed that SCB was more effective in modulating the production of the CAZymes required for the degradation of SCB than the MCC and wheat bran present in the cultivation media.

**Fig. 4:**
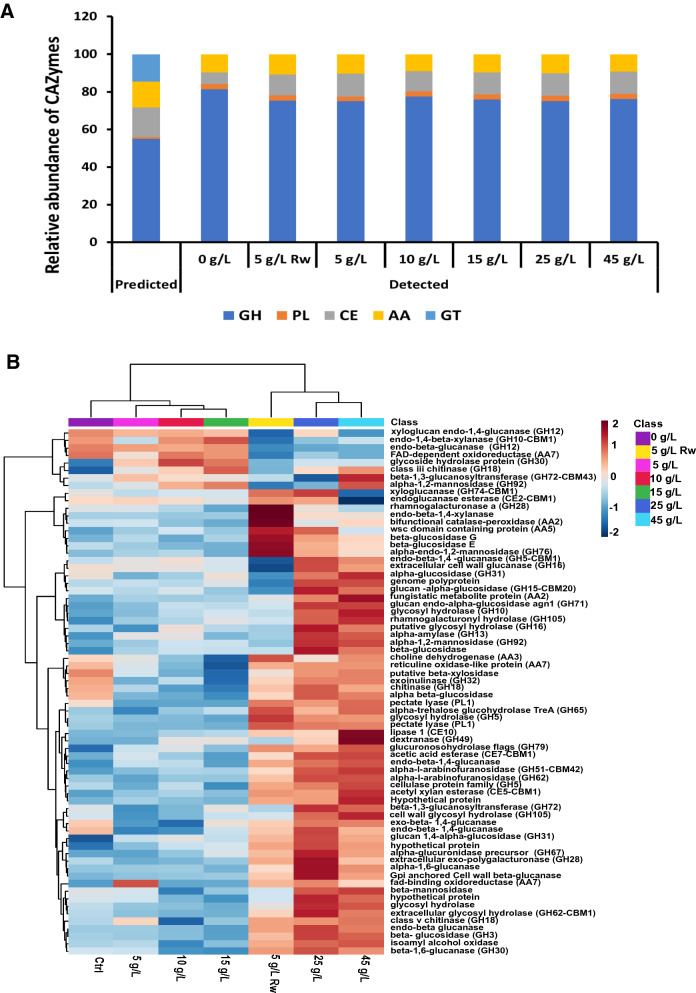
**A** Number and distribution of predicted CAZymes obtained from the draft genome sequence versus CAZymes detected in secretomes of *Penicillium funiculosum* (*Pf*Mig1^88^) modulated with sugarcane bagasse. Values in each category represent the actual number of CAZymes predicted and detected. *GT* glycosyl transferases, *AA* auxiliary activities, *CE* carbohydrate esterases, *PL* polysaccharide lyases, *GH* glycoside hydrolases. **B** Hierarchical clustering of proteins present in the different secretomes of *Pf*Mig1^88^ showing the abundance of differentially associating proteins. Ctrl refers to the control sample without sugarcane bagasse (0 g/L) while Rw refers to untreated sugarcane bagasse

Figure 4 shows a comparative evaluation between all possible CAZymes from the draft genome sequence and those detected in the secretomes of *Pf*Mig1^88^ under different cultivation conditions. Also, a hierarchical clustering to give a visual representation of the distribution and the relative abundance of the major proteins detected across the different secretomes is given in Fig. 4B. The expression level of various CAZy classes in the secretomes induced with SCB showed a gradual increase in the relative abundance of carbohydrate esterases (CE) required to de-esterify hemicellulosic sugars. While there were no changes in the relative abundances of the proteins with auxiliary activities (AAs) and polysaccharide lyase (PLs), a gradual reduction in the relative abundance of glycoside hydrolases (GHs) in all the secretomes was also observed (Fig. 4A). The changes in the proportion of CEs across the secretomes containing bagasse could be attributed to the nature of the complexity of the sugarcane bagasse introduced into the production media, requiring more cell wall-modifying enzymes such as the esterases in order to render it more accessible to the GHs for hydrolysis of hemicellulose and cellulose [22]. The hierarchical clustering data showed clustering of proteins of 5, 10 and 15 g/L pretreated SCB in one branch near to control, while proteins of 25 and 45 g/L pretreated SCB sit in a different branch along with the 5 g/L raw SCB (Fig. 4B).

### Comparative regulation dynamics of secreted proteins in response to sugarcane bagasse by *Pf*Mig1^88^

**Fig. 5:**
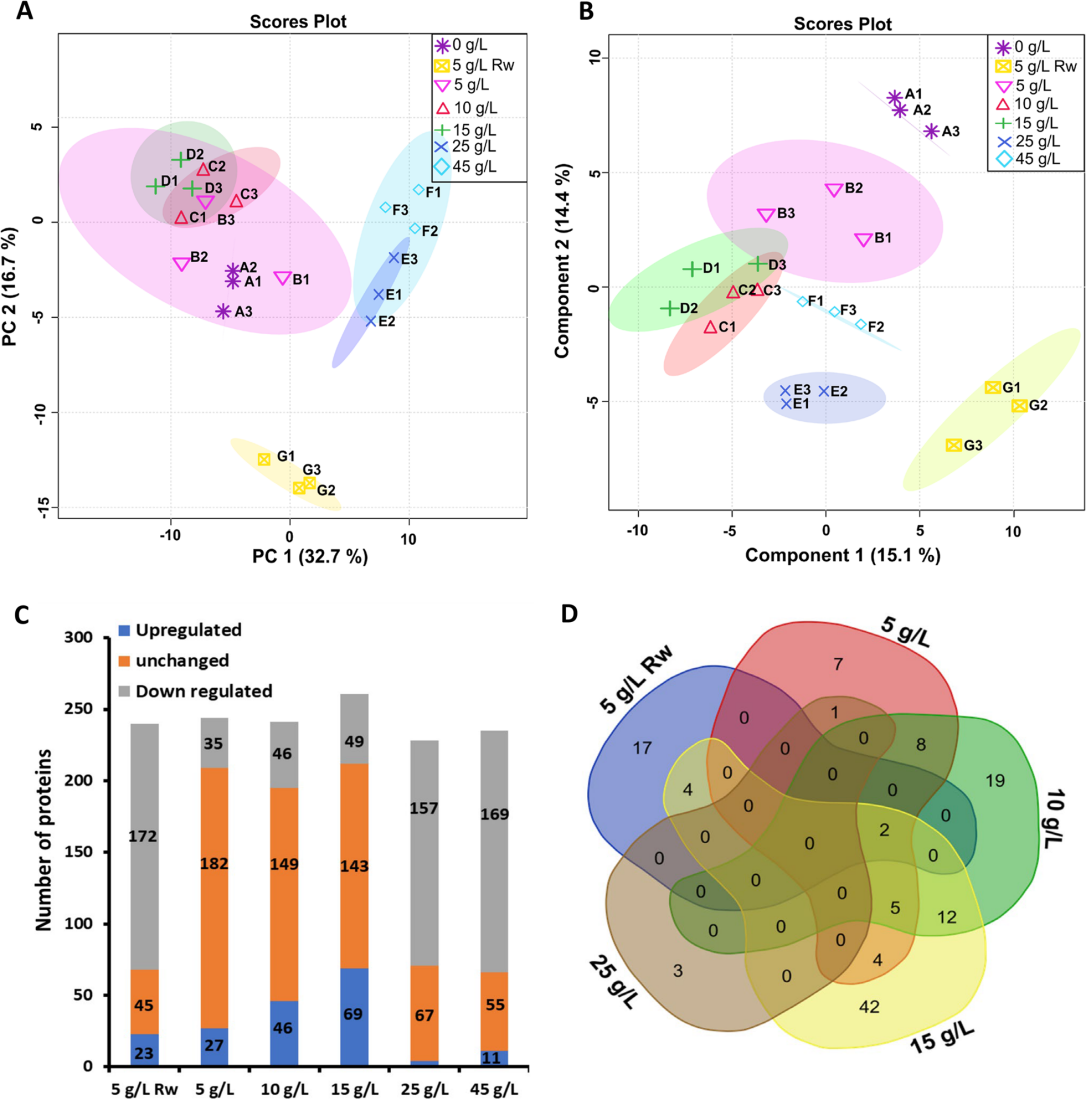
Substrate-induced variations in *P. funiculosum* (*Pf*Mig1^88^) response to pretreated sugarcane bagasse relative to control without bagasse. **A** Principal component analysis (PCA) and **B** partial least squares-discriminant analysis (PLS-DA) of proteomic data obtained from *Pf*Mig1^88^ secretomes induced by different concentrations of pretreated sugarcane bagasse. **C** Regulation number of lignocellulases and other secreted proteins induced by pretreated sugarcane bagasse in comparison to control (without bagasse). **D** Distribution of substantially regulated proteins across secretomes of *Pf*Mig1^88^ secretomes induced by different concentrations of pretreated sugarcane bagasse. Rw refers to untreated sugarcane bagasse

In order to gain further insight into the interaction and regulation of the various CAZymes secreted by *Pf*Mig1^88^ under different induction conditions with SCB, we performed an in-depth statistical analysis on the abundance of each protein across the different secretomes. Through PCA and PLSDA analysis (Fig. 5A, B), we found a close similarity between the proteins produced in the presence of 5, 10 and 15 g/L of pretreated SCB. These are characterized by the presence of core cellulases and hemicellulases, such as endo-1,4-β-glucanase (GH7-CBM1), cellobiohydrolase I (GH7-CBM1), endo-1,4-β-xylanase (GH30-CBM1), α-1,6-glucanohydrolase (GH 49) and endo-1,4-β-mannosidase (GH5). Furthermore, lignin-depolymerizing enzymes such as catalase-peroxidase (AA2), catalase B, FAD-dependent oxidase (AA7) and choline dehydrogenase (AA3) were also identified in these secretomes. The presence of these lignolytic enzymes could be attributed to lignin retained in the SCB used for supplementing the production media [23]. The modulation of the production media with raw and higher concentrations of pretreated SCB affected the distribution of the proteins secreted. The secretomes supplemented with higher concentrations of SCB (25 and 45 g/L) showed differences as compared with those with lower concentrations with increased abundance of enzymes involved in cell wall organization such as endo-polygalacturonase (GH28), GPI-anchored cell wall beta-1,3-endoglucanase (GH17) and acetyl xylan esterase (CE5-CBM1). The proteins produced by *Pf*Mig1^88^ induced with raw SCB were very different to those produced by the fungus supplemented with pretreated bagasse and characterized by the abundance of bifunctional catalase-peroxidase (AA2), endo-1,4-beta-mannosidase (GH5), endo-α-mannosidase (GH76), a putative GH16 glycosyl hydrolase protein, aminopeptidase 2 and two hypothetical proteins (Fig. 5B, Additional file 1: Table S2). This is possibly due to the different composition of the biomass before and after pretreatment, with different lignin and cellulose content (Fig. 1A).

The different secreted proteins across all the secretomes supplemented with SCB were studied by determining the abundance ratio of each of the detected proteins across all the secretomes (Additional file 2). Proteins with abundance ratio ≥ 2 were considered to be upregulated while those with abundance ratio of 1 were considered unchanged and those below 1 were downregulated. A summary of the statistics showing the distribution of the upregulated and downregulated proteins, as well as those unchanged across the secretomes is presented in Fig. 5C. Interestingly, the results showed a progressive increase in the number of upregulated proteins with increasing concentration of SCB until 15 g/L. However, most of the proteins present in the secretomes supplemented with 25 and 45 g/L SCB were highly downregulated with the majority of them being proteases and cell wall-associated proteins (Fig. 5C). The decrease in the number of essential enzymes required for biomass deconstruction in these secretome could be due to the inhibitors generated during the pretreatment process having an adverse effect on the fungus growth and limiting the production of cellulolytic enzymes. A similar result was reported earlier for the parent strain of this fungus (NCIM122), where the majority of the proteins secreted in response to complete replacement of the carbon source with pretreated wheat straw were enzymes produced in response to oxidative stress [13]. Also, the presence of highly recalcitrant lignin in the untreated SCB could have accounted for the higher number of downregulated proteins in the secretome supplemented with 5 g/L of untreated SCB.

**Fig. 6:**
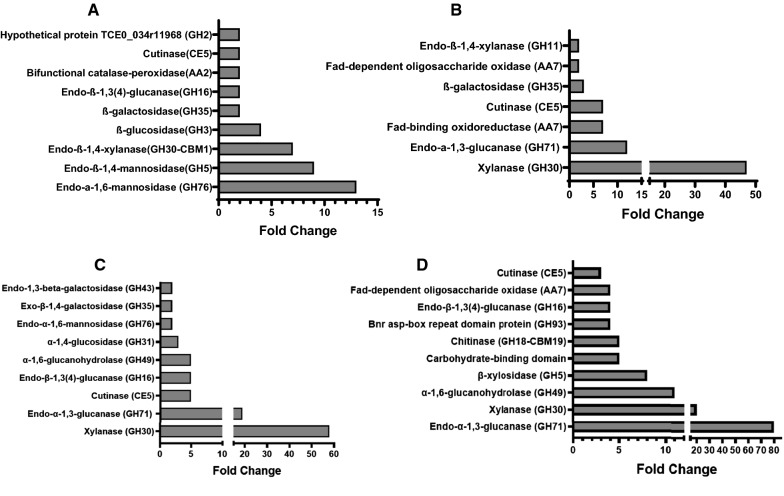
Highlight of the top most abundant and sample specific proteins present in the secretome of *Pf*Mig1^88^ modulated with pretreated sugarcane bagasse. **A** Media supplemented with 5 g/L untreated SCB (Rw). **B** Media supplemented with 5 g/L pretreated SCB. **C** Media supplemented with 10 g/L pretreated SCB. **D** Media supplemented with 15 g/L pretreated SCB. The fold change in protein expression were calculated from the log2-fold ratio obtained from the mass spectrometry analysis

An overview of the distribution of the upregulated protein across the SCB-supplemented secretomes is shown in Fig. 5D and Additional file 1: Table S2. We found a total of 126 upregulated proteins and the most upregulated proteins under each test conditions (5–15 g/L SCB) are presented in Fig. 6. The results showed remarkable induction of xylanase (GH30) and endo-α-1,3-glucanase (GH71) with increased concentration of SCB. The increase in abundance level of xylanase found with increased concentration of pretreated SCB could be attributed to the nature of the biomass which is substantially rich in glucuronoarabinoxylan, a crucial inducer of this important enzyme for biomass deconstruction [24]. Also, the high induction of endo-α-1,3-glucanase in the secretomes with increased SCB suggests the presence of some mixed glucans in the bagasse which could have facilitated the induction of the enzyme. Furthermore, there was significant upregulation of cutinase (CE5) and β-galactosidase (GH35) in the secretomes supplemented with raw and pretreated SCB up till 15 g/L. Cutinase, a carbohydrate esterase family 5 protein is an inducible lipolytic/esterolytic enzyme, capable of catalyzing the cleavage of the ester bonds of cutin and insoluble triglycerides facilitating fungal penetration to the biomass through the cuticle [25, 26]. Cutinase has been reported as an important accessory enzyme for enhancing the hydrolysis of lignocellulosic biomass for second-generation biofuel production asides other industrial applications [27–30]. This enzyme works in synergy with hydrophobins (HsbA) in the sensing of physical association with hydrophobic surfaces and promotion of substrate degradation by recruiting hydrolases to the surface of lignocellulosic biomass [11]. Previous transcriptomics studies have also shown significant upregulation of cutinase gene from *Aspergillus niger* and *Trichoderma harzianum* IOC3844 when cultivated in the presence of SCB suggesting it as an important accessory enzyme for the deconstruction of SCB [31, 32]. β-Galactosidase (GH35) is another important pectin-degrading enzyme that facilitates the degradation of pectin in the cell wall of SCB. Secretomes with 10 and 15 g/L SCB showed the highest number of common upregulated proteins, mainly β-xylosidase (GH5, GH43), β-1,3-galactosidase (GH35, GH43); hydrophobic surface-binding protein (HsbA) and endo-β-1,3(4)-glucanase (GH16) responsible for hemicellulose degradation (Additional file 1: Table S2). While most of the essential cellulases required for cellulose deconstruction showed a minimal change in the presence of pretreated SCB at 5–15 g/L, they were highly downregulated in the presence of 5 g/L raw, 25 g/L and 45 g/L of pretreated bagasse (Table 2). This may probably be a result of the replacement of crystalline cellulose in the production media with higher amounts of pretreated SCB, emphasizing the important role of crystalline cellulose in the induction of carbohydrate-degrading enzymes by *P. funiculosum* as reported in our previous work [13]. Also, the nature of the recalcitrance of the raw SCB could have also triggered the cellular machineries to secrete different kind of enzymes asides the cellulolytic enzymes thereby accounting for the high downregulation as seen earlier in Fig. 5A, B.

**Table 2:** Regulation of key cellulolytic enzymes present in *Pf*Mig1^88^ secretomes modulated with SCB

Accession	Description	CAZY	log2 (fold change)					
			5 g/L raw	5 g/L	10 g/L	15 g/L	25 g/L	45 g/L
maker-contig00012-exonerate_protein2genome-gene-4.113-mRNA-1	Cellobiohydrolase I	GH7-CBM1	− 5.72	− 0.23	− 0.21	0.14	− 5.88	− 5.72
maker-contig00037-exonerate_protein2genome-gene-1.53-mRNA-1	Cellobiohydrolase II	GH6-CBM1	− 1.18	− 0.25	− 0.12	− 0.87	− 0.63	− 0.45
maker-contig00035-exonerate_protein2genome-gene-2.46-mRNA-1	Endo-β-1,4-glucanase	GH5-CBM1	− 0.94	0.07	0.20	0.18	− 0.31	− 1.11
maker-contig00064-exonerate_protein2genome-gene-0.26-mRNA-1	Endo-β-1,4-glucanase	GH7-CBM1	− 6.64	− 0.26	− 0.29	− 0.10	− 6.64	− 6.64
maker-contig00040-exonerate_protein2genome-gene-0.38-mRNA-1	β-Glucosidase	GH3	− 6.16	− 0.19	− 0.10	− 0.06	− 4.27	− 4.92
maker-contig00069-exonerate_protein2genome-gene-0.5-mRNA-1_1	β-Glucosidase	GH3	ND	− 0.27	− 0.27	− 0.62	− 2.16	− 1.47
maker-contig00104-exonerate_protein2genome-gene-0.20-mRNA-1	Endo-β-1,4-xylanase	GH30-CBM1	2.87	− 0.94	0.46	1.08	− 0.39	− 0.68
maker-contig00004-exonerate_protein2genome-gene-6.48-mRNA-1	Lytic polysaccharide monooxygenase	AA9	− 4.38	− 0.62	− 1.60	− 0.73	− 3.28	− 3.76

ND refers to non-detected

### Functional analysis of essential biomass-degrading enzymes in the secretomes of *Pf*Mig1^88^

**Fig. 7:**
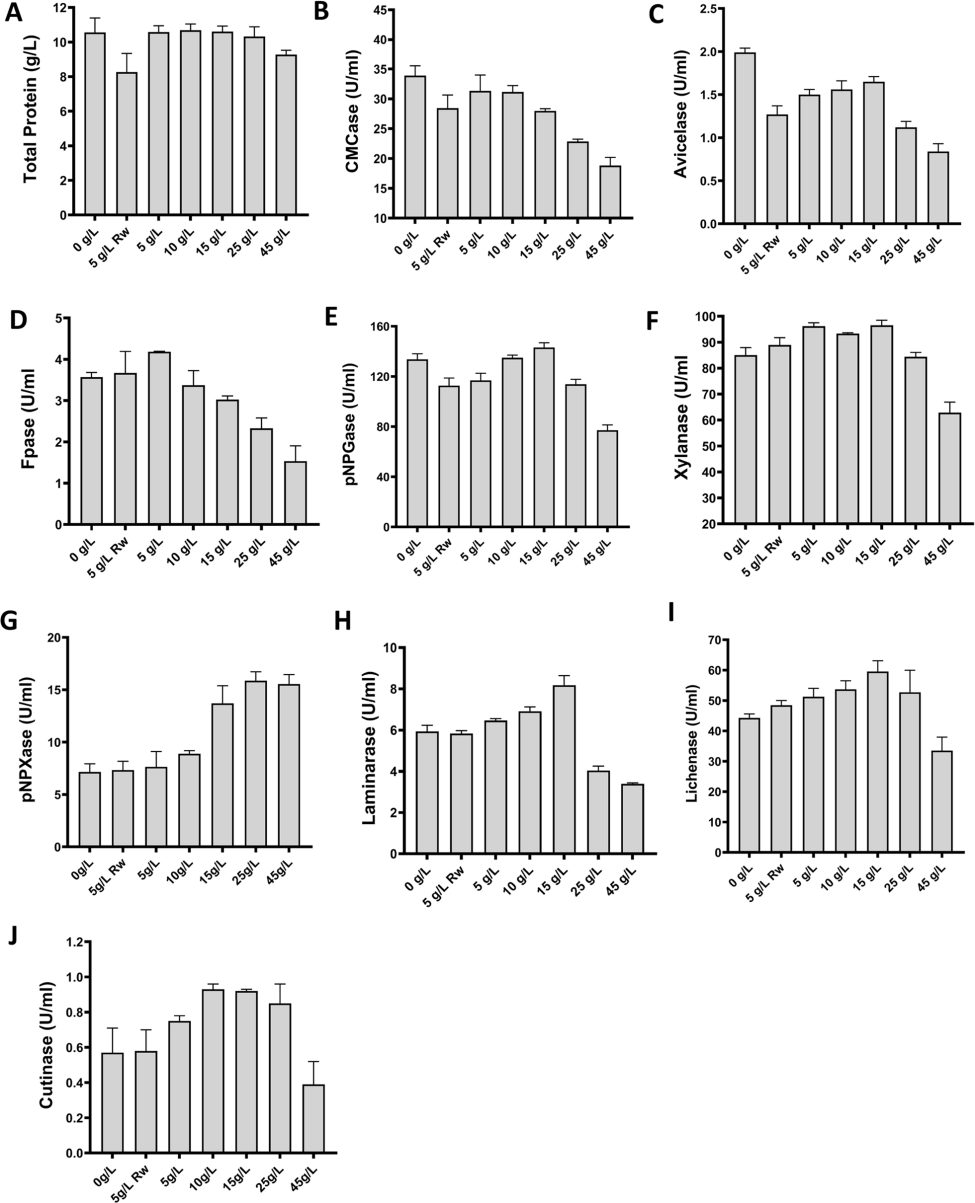
Enzymatic activities and total protein concentration in *Pf*Mig1^88^ secretomes induced by raw and different concentrations of pretreated sugarcane bagasse as carbon sources. **A** the total secreted proteins in all the secretomes as determined by BCA; **B** the endoglucanase activity determined using CMC as the substrate; **C** the CBH1 activity determined using Avicel as the substrate; **D** the overall cellulase activity on filter paper; **E** β-glucosidase activity determined using pNPG; **F** xylanase activity determined using beechwood xylan; **G** β-xylosidase activity determined using pNPX. **H** Laminarase activity determined using laminarin as substrate; **I** Lichenase activity determined using lichen as substrate; **J** cutinase activity determined using pNPB. The data are presented as the means of three independent experiments, and error bars express the standard deviations. Rw refers to untreated sugarcane bagasse

To assess the enzymatic repertoire of *Pf*Mig1^88^ for the deconstruction of SCB, a comprehensive analysis of key enzymes with known functions in deconstruction of polysaccharides was carried out. The hydrolytic performance of these enzymes was assessed biochemically using different substrates with the secretomes from cultures under all the conditions described above. As expected from the proteomics results, there was no significant difference in the amount of total protein secreted by the fungus as a result of the supplementation of the production media with pretreated SCB. An exception was the culture grown in the presence of raw SCB, which had lower protein concentration (Fig. 7A). The activities of the individual cellulose deconstructing enzymes showed a reduction in endoglucanase (CMCase) and cellobiohydrolase (Avicelase) activities after the replacement of crystalline cellulose in the production media as compared to control (Fig. 7B, C). This led to an overall decrease in the total cellulase activity measured using the filter paper assay at higher SCB concentration (Fig. 7D). There was no significant change in β-glucosidase activity of the different secretomes, except at the highest concentration of SCB (Fig. 7E). These results were consistent with the mass spectrometry data where most of the essential cellulose-degrading enzymes were downregulated in the course of replacing crystalline cellulose with SCB (Table 2). However, in contrast to the cellulose-degrading enzymes, we noticed that the gradual supplementation of the production media with SCB enhanced the activities of the hemicellulases secreted. Xylanase activity in the media containing raw SCB increased by 5% while those with pretreated SCB (5–15 g/L) increased by 14% (Fig. 7F). Interestingly, we found a substantial increase in the β-xylosidase activity of the fungus (in the range of 7–122%) with increasing concentration of SCB in the production media (Fig. 7G). The production media with 25 g/L SCB produced the maximum activity of β-xylosidase (15.9 U/mL), 122% higher than the control without SCB (7.7 U/mL). The maximum yield obtained was similar to that reported for a β-xylosidase overexpressing strain of *Penicillium oxalicum* RGXyl-1, where the maximum was 15.1 U/mL [33] and *Penicillium sclerotiorum* with a β-xylosidase activity of 17.5 U/mL [34]. This result shows that the adjustment of the medium composition with SCB enhanced the secretion of this crucial lignocellulolytic enzyme and suggests that the titer could be further improved upon overexpression. Similarly, activities on mixed-linked glucan substrates, which are part of the hemicellulose fraction in sugarcane cell walls were induced by SCB supplementation (Fig. 7H, I). While there was progressive increase in lichenase and laminarase activities with increasing concentration of SCB, the maximum activities were obtained with secretome containing 15 g/L SCB (Fig. 7H, I). These results were in agreement with earlier reports, where SCB induced the secretion of these industrially relevant enzymes [21, 35, 36]. This is potentially relevant since enzymatic cocktails required for deconstruction of lignocellulosic biomass such as SCB should be supplemented with β-(1,4)-, β-(1,3)-, and β-(1,6)-glucanases due to the presence of β-(1,3)- and β-(1,4)-d-glucans in plant cell wall [37, 38]. While previous reports have shown that the expression of cutinase by filamentous fungi could be induced by SCB, there are no reports of expression at the protein level in the presence of SCB. Since this enzyme was highly upregulated in all the secretomes produced in the presence of SCB (Fig. 6), its activity towards *p*-nitrophenyl butyrate (pNPB) was evaluated (Fig. 7J). Cutinase was induced between 30 and 60% by supplementation of the production media with SCB, suggesting a potential role in improving the saccharification performance of the *Pf*Mig1^88^ secretome on SCB.

### Saccharification dynamics of pretreated sugarcane bagasse by *Pf*Mig1^88^ secretomes

**Fig. 8:**
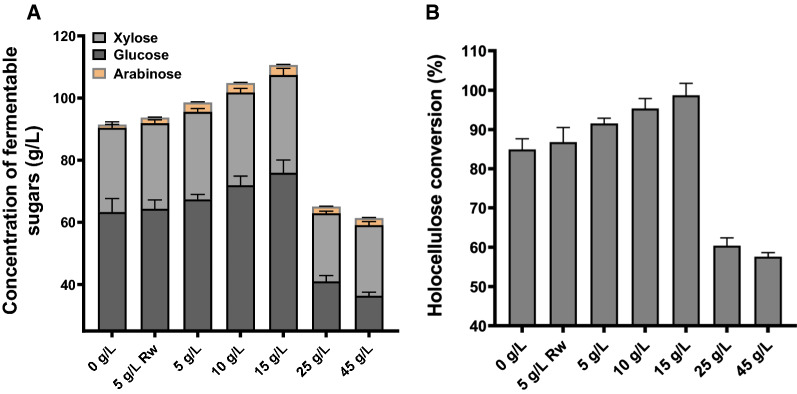
Saccharification of pretreated sugarcane bagasse by the secretomes of *Pf*Mig1^88^ under 15% solid loading and a protein concentration of 7 FPU/g biomass. **A** Total fermentable sugar obtained at the 72 h saccharification period. **B** Percentage holocellulose conversion measured at 72 h saccharification period. Rw refers to untreated sugarcane bagasse

After identifying the array of enzymes upregulated in the secretome of *Pf*Mig1^88^ in response to the supplementation of the culture media with varying concentrations of the bagasse, we evaluated the saccharification potential of the secretomes on pretreated SCB. For this, the saccharification reaction was set up using a 15% substrate loading and a cocktail loading of 7 FPU/g dry biomass weight (DBW)_,_ and incubated at 50 °C for 72 h. The results showed a gradual increase in the amount of reducing sugars released and saccharification efficiency with increasing concentrations of SCB up to 15 g/L, after which there was a decline compared with the secretome produced without SCB (Fig. 8). The highest holocellulose (cellulose + hemicellulose) conversion yields were achieved with secretomes containing 10 g/L and 15 g/L SCB, exhibiting ~ 15% and 20% increase, respectively, over the secretome without SCB. The observed increase in the yield of monomeric sugars (glucose, xylose and arabinose) obtained with the introduction of SCB in the cultivation media could be attributed to increased activity of the hemicellulases present in the secretomes and in agreement with the increase in the activities of hemicellulases (lichenase, β-xylosidase, laminarase). This implied that the increased hydrolysis of the hemicellulose component of the bagasse by the hemicellulases complemented the action of the cellulases by exposing the cellulose component to enzymatic attack, as previously reported [33, 39, 40]. In addition, the hydrolysis of the lipid component of the bagasse such as the insoluble triglycerides or polyesters by cutinase may have also provided greater access to endoglucanases, endoxylanases and pectate lyases [28, 30]. It is interesting that the induction and secretion of these enzymes as seen in this study could have contributed to the saccharification of SCB to monomeric sugars which can subsequently be converted to important value-added products such as citric acid, bioethanol and many others. Overexpression of these important enzymes could subsequently be employed to complement the induction of the enzymes in the fungus secretome as observed here. Also, detailed biochemical characterization of some of the specific enzymes from this strain will contribute to understanding their properties for application in an industrial context.

## Conclusion

In this study, the systematic evaluation and quantification of enzymes produced by this engineered fungus during growth on pretreated SCB provides an insight into the secretome dynamics and regulation of the major proteins for efficient SCB saccharification. The secretome analyses indicated which enzymes may be key for hydrolysis and act synergistically for efficient deconstruction. We found CAZymes distributed across different subfamilies (GH3, GH5, GH6, GH7, GH10, GH11, GH16, GH30, GH43, GH62, GH71, GH93, CE1, CE5, AA7 and AA9) that were secreted and played important roles in biomass degradation. Most importantly, some essential hemicellulases and accessory enzymes such as endo-β-1,3(4)-glucanase (GH16), endo-α-1,3-glucanase (GH71), xylanase (GH30), β-xylosidase (GH5) and cutinase (CE5) were identified in the *Pf*Mig1^88^ secretome induced with SCB. This suggests that these enzymes could be potential targets for rational engineering of an optimized fungal-derived enzyme cocktail.

## Materials and methods

### Chemical pretreatment of sugarcane bagasse and its compositional analysis

SCB was kindly provided by Natems Sugars Private Limited, Hyderabad, Telangana, India. SCB was washed, dried in a convection oven at 60 °C to constant weight, and then knife-milled using a 1 mm sieve in the mill. The alkaline pretreatment of SCB was done at 90 °C using 0.4 M NaOH at 10% solid loading for 6 h. After pretreatment, the solids were washed three times and their pH was lowered to 6.0 using H_2_SO_4_. Lignin content was determined by the acetyl bromide method in the samples before and after alkaline pretreatment [41]. The absorbance was measured at 280 nm in triplicates using the extinction coefficient for grasses (17.75 L/g cm) and expressed as percentage of lignin on a dry weight basis [42].

For the determination of hemicellulose and cellulose polysaccharides, dry material (4 mg) was hydrolyzed with 0.5 mL of 2 mol/L trifluoroacetic acid (TFA) for 4 h at 100 °C under argon atmosphere. After hydrolysis, TFA was eliminated by evaporation and subsequent washes with isopropanol. Samples were resuspended in water and the monosaccharides released were analyzed by high-performance anion-exchange chromatography with pulsed amperometric detection (HPAEC-PAD) as previously reported [43]. The fraction released by TFA was considered as hemicellulose. Crystalline cellulose determination was performed by hydrolysis of the remaining solid fraction with 90 μL of 72% H_2_SO_4_ (w/w) at 25 °C for 4 h under argon atmosphere [44], followed by incubation at 120 °C. Glucose content was determined by the colorimetric anthrone–sulfuric acid assay [45]. Cellulose content was determined in triplicate and expressed as the percentage on a dry weight basis.

### Microorganism, cultivation conditions with sugarcane bagasse and secretome extraction

*Penicillium funiculosum* Mig1^88^ (*Pf*Mig1^88^), a catabolite derepressed strain of *P. funiculosum* NCIM1228, was used for this study. The strain was cultivated on Petri dishes containing low malt extract agar until there was full sporulation. Conidiospores from it were thereafter recovered with sterile water, filtered through sterile Miracloth and quantified using a haemocytometer. The primary culture was prepared by inoculating 10^7^ conidiospores/mL in potato dextrose broth (PDB) and incubated at 28 °C for 36 h before being used to inoculate secondary media. For enzyme production, the *Pf*Mig1^88^ strain was periodically cultivated in 250 mL Erlenmeyer flasks containing 50 mL of an optimized cellulase-inducing media (CIM) comprising soya peptone (24 g/L), wheat bran (21.4 g/L), microcrystalline cellulose [MCC (24 g/L)], KH_2_PO_4_ (12.4 g/L), K_2_HPO_4_ (2.68 g/L), (NH4)_2_SO_4_ (0.28 g/L), CaCO_3_ (2.5 g/L), corn steep liquor (1%), urea (0.52 g/L), and yeast extract (0.05 g/L) with the final pH adjusted to 5.0 as previously determined [12]. To evaluate the effect of SCB on enzyme secretion by *Pf*Mig1^88^, the proportion of carbon source in the production media was systematically modified with raw and pretreated SCB. The MCC and wheat bran content in the cellulase-inducing media were modulated using both raw and NaOH pretreated SCB at different concentrations ranging from 0 to 45 g/L as shown in Table 3 in order to compare their ability to induce the production of lignocellulolytic enzymes specific for its deconstruction. The flasks were incubated at 28 °C for 5 days with orbital shaking at 150 rpm (Innova 44, Eppendorf AG, Germany). Induced cultures were centrifuged at 9000 rpm for 10 min at 4 °C. All experiments were performed in triplicate. The resulting supernatants were collected and used for different enzymatic assays as well as for proteomics studies.

**Table 3:** Experimental setup for modulation of the cellulosic substrates used in production media for cultivation of *Pf*Mig1^88^

Sugarcane bagasse (g/L)	Microcrystalline cellulose (g/L)	Wheat bran (g/L)	Total (g/L)
0	24	21	45
5	21.2	18.8	45
10	18.6	16.4	45
15	15.9	14.1	45
25	10.6	9.4	45
45	0	0	45

### Enzyme assays and total protein quantification

All enzymatic activities performed in this study were routinely determined following standard assay procedures. Cellobiohydrolase activity was determined by incubating appropriate dilution of the enzyme with 1% Avicel® PH-101 (Sigma) for 120 min. Endoglucanase, xylanase, lichenase, laminarase, β-glucosidase and β-xylosidase activities were determined by incubating appropriate dilution of the enzyme with 2% CMC (Sigma), 2% beechwood xylan (HiMedia), 1% lichenan (Megazyme), 1% laminarin (Sigma), *p*-nitrophenyl-β-d-glucopyranoside (Sigma) and *p*-nitrophenyl-β-d-xylopyranoside (Megazyme), respectively, for 30 min, after which the amount of reducing sugars released was measured as previously reported [12]. One unit of CMCase, Avicelase, xylanase, lichenase and laminarase activity is defined as the amount of enzyme releasing 1 µmol of reducing sugar per min while one unit of β-glucosidase and β-xylosidase activities was defined as the amount of protein that released 1 μmol of *p*-nitrophenol (pNP) per min. Total cellulase activity in the secretome was measured in terms of filter paper units (FPU) per milliliter of original (undiluted) enzyme solution. The assay requires a fixed degree of conversion of substrate, from 50 mg of filter paper within 60 min at 50 °C. One FPU is defined as the amount of enzyme required to produce 2 mg of glucose from 50 mg of filter paper within 60 min of incubation. Cutinase activity was assayed by the method described by Duan et al. [27] using pNPB as a substrate. The reaction mixture (500 μL) contained 400 μL 50 mM Tris–HCl buffer pH 8.0, 50 μL suitably diluted enzyme, and 50 μL of 20 mM pNPB dissolved in isopropanol. After 10 min at 50 °C, the released pNP was quantified by the absorbance at 410 nm. One unit of enzyme activity was defined as the amount of enzyme liberating 1 μmol pNP per min under the above conditions. To quantitate the amount of secreted proteins in the various *Pf*Mig1^88^ secretomes, the crude secretomes were first buffer exchanged with the help of 10-kDa cut-off membrane using citrate–phosphate buffer (pH 4.0) and then the total protein of each secretome was estimated by the bicinchoninic acid (BCA) method using bovine serum albumin (BSA) as standard.

### Protein preparation for proteomics experiments

To prepare the samples for proteomic studies, secretome aliquots containing 50 µg of total protein were precipitated following the method described by Crowell et al. [46]. The precipitated protein was then vacuum-dried, reconstituted in 150 µL of resuspension buffer containing 8 M urea, 7.5 mM NaCl, 50 mM tri-ethylammonium bicarbonate (TEAB) pH 8.2, 10 mM dithiothreitol (DTT), and incubated at 56 °C for 45 min as described by Ogunmolu et al. [11]. This was followed by the addition of 20 μL of 200 mM iodoacetamide prepared in 50 mM TEAB and incubated at 25 °C in the dark for 60 min. DTT was then added to a final concentration of 10 mM to consume any unreacted iodoacetamide and the reaction mixture was further incubated for additional 60 min in the dark. After incubation, the reaction mixture was diluted fivefold in 50 mM TEAB, pH 7.6, followed by the addition of CaCl_2_ to a final concentration of 1 mM. Samples were then digested using sequencing grade trypsin (1 µg per 50 µg of total protein; Pierce Biotechnology, USA) overnight at 37 °C. The enzymatic digestion was terminated by the addition of formic acid to pH 3.0 to 4.0. The tryptic peptides were desalted using C18 spin columns (Thermo Fisher Scientific). Eluted samples were vacuum-dried and reconstituted in 0.1% (v/v) formic acid before being subjected to LC–MS/MS.

### Data acquisition, processing and analysis

LC–MS/MS analysis was performed using Orbitrap Fusion Lumos Tribrid Mass Spectrometer equipped with nano-LC Easy nLC 1200 (Thermo Fischer Scientific, Singapore). Liquid chromatography separation was performed at a flow rate of 300 nL/mL on a C18 pre-column (Acclaim PepMap™ 100, 75 µm × 2 cm, nanoViper, P/N 164946, ThermoFisher Scientific Incorporation) followed by analytical column (Acclaim PepMap™ RSLC C18, 75 µm × 50 cm, 2 µm, 100 Å, P/N ES803). The peptides were separated using a gradient of 2% solvent B to 10% in 5 min followed by gradient increase to 45% and sharp increase to 95%, then retention of 95% for 10 min. Solvent A was aqueous solution in 0.1% formic acid, and solvent B was 95% acetonitrile in 0.1% formic acid. The eluted peptides were injected into the mass spectrometer and the MS1 data were acquired in full scan mode at 120,000 orbitrap resolution with mass range from 375 to 2000 Da. Data were acquired using the Thermo Xcalibur software setup version 4.3.73.11 (Thermo Fischer Scientific, Inc 2019). Precursors were allowed to fragment using Higher-energy C-trap dissociation (HCD) in ion trap (IT) detector with collision energy of 28 in a data dependent MS^n^ Scan acquisition. Charge state screening of precursor ions and monoisotopic precursor selection was enabled. The parent ions once fragmented were excluded for 40 s with exclusion mass width of ± 10 ppm. The lock mass option (polydimethylcyclosiloxane; m/z 445.120025) enabled accurate mass measurement in the MS Mode. For analysis, raw LC–MS/MS data files obtained from the mass spectrometer were processed with Proteome Discoverer™ (Version 2.4.1.15, Thermo Fisher™ Scientific Inc.). The Proteome Discoverer processing workflow was employed in the label-free quantitation (LFQ) of relative protein abundance across the samples and controls. For the search process, Mascot and Sequest HT tools were used. Peak lists obtained from MS/MS spectra were identified using the MSF files. Protein identification was conducted against a concatenated target/decoy version of the in-house predicted proteins or in-house database (11,213 target sequences) obtained from the draft genome sequence of *Penicillium funiculosum* along with Proteome Discoverer contaminant database. The identification settings were as follows: trypsin digestion with maximum of 2 missed cleavages; minimum peptide length 6; precursor mass tolerance 20 ppm; fragment mass tolerance 0.5 Da; fixed modifications; carbamidomethyl c (+ 57.021464 Da), variable modifications; oxidation of m (+ 15.994915 Da), acetylation of protein n-term (+ 42.010565 Da). Peptides and proteins inferred from the spectrum results using SwissProt and Uniprot database. Peptide Spectrum Matches (PSMs), peptides and proteins were validated at a target False Discovery Rate (FDR) strict to 0.01 and relaxed to 0.05. The relative protein abundance estimation including normalization, hierarchical cluster analysis, scatter plots were performed using Proteome Discover LFQ workflow. Venn representations of protein were done using http://bioinformatics.psb.ugent.be/webtools/Venn/. Function assignment and annotation by gene ontology terms (GO; www.geneontology.org), InterPro terms (InterProScan, EBI) and enzyme classification codes (EC) were determined using the Blast2GO suite [47]. Annotations were made with default parameters and were augmented by using the Annotation Expander (ANNEX) and by the addition of the GO terms associated with functional domains derived from scanning the InterPro database. Carbohydrate and auxiliary-active enzyme families were assigned using the CAZy database—http://www.cazy.org. Normalized protein abundance values of all the secretomes containing SCB (5–45 g/L) were compared to the secretome without SCB by ANOVA (*p* < 0.05) and student’s *t* test (*p* < 0.05). To evaluate the protein distribution pattern under each of the test conditions, data were subjected to principal component analysis (PCA) and partial least squares-discriminant analysis (PLS-DA) using metaboAnalysis platform [48].

### Saccharification of pretreated sugarcane bagasse by *Pf*Mig1^88^ secretomes

The saccharification efficiency of the secretomes of *Pf*Mig1^88^ produced in response to different concentration of pretreated SCB was evaluated at 15% solid loading in 50 mM sodium citrate phosphate buffer (pH 4.0) and 7 FPU/g of different *Pf*Mig1^88^ secretomes. Saccharification was performed in 50 mL screw-capped Falcon tubes with a working volume of 5 mL in an incubator shaker at 50 °C and 200 rpm for 72 h. Control experiments were carried out under the same conditions using substrates without enzymes (enzyme blank) and enzymes without substrates (substrate blank); a substrate-free negative control was set up by filling the Falcon tubes with 100 mM citrate–phosphate buffer, pH 4.0, and the background of soluble sugars present in the respective biomass was determined by incubating each biomass in the absence of enzyme. Following the completion of hydrolysis at each time point, the Falcon tubes were centrifuged at 3500 rpm for 10 min in a swinging bucket centrifuge (Eppendorf, Germany) to separate the solid residue from the digested biomass. Supernatants which were recovered after enzymatic hydrolysis of the pretreated SCB were analyzed by high-performance liquid chromatography equipped with Aminex HPX-87H anion exchange column (Bio-Rad, USA) and a refractive index detector to analyze released monosaccharides (glucose and xylose) by anion-exchange chromatography. The filtered mobile phase (4 mM H_2_SO_4_) was used at a constant rate of 0.3 mL/min with the column, and RI detector temperatures maintained at 35 °C. The concentration of each monosaccharide was calculated from calibration curves of external standards (xylose and glucose) purchased from Absolute Standards Inc. The theoretical conversions of cellulose and hemicellulose (in percentage) into monomeric sugars were calculated using the equations provided in NREL's LAP TP-510-43630 as earlier reported [12, 49].

### Data and statistical analysis

All experiments were performed in triplicate, and the results are presented as the means and standard deviations. The data were compiled in a Microsoft Excel spreadsheet, where the averages and standard errors of the means were determined. All graphs were created using GraphPad Prism 8.0 software.

## Supplementary Information

**Additional file 1: Table S1.** The functional annotations of proteins identified in the proteome of* Pf*Mig1^88^. **Table S2.** The distribution of upregulated proteins across the different secretomes containing sugarcane bagasse.

**Additional file 2: **Total number of proteins detected in the proteome of* Pf*Mig1^88^ modulated with varying concentrations of sugarcane bagasse and their abundance ratio.

## Data Availability

The mass spectrometry proteomics data for this study have been deposited to the ProteomeXchange Consortium using the PRIDE [50] partner repository with the dataset identifiers PXD023063 and 10.6019/PXD023063.
