# Supplementary material for: Accessory enzymes of hypercellulolytic Penicillium funiculosum facilitate complete saccharification of sugarcane bagasse
Source: Biotechnol Biofuels. 2021 Aug 26;14:171. doi: 10.1186/s13068-021-02020-x (PMC8394629; doi:10.1186/s13068-021-02020-x)
Supplement: Supplementary file 1 — Additional file 1: Table S1. The functional annotations of proteins identified in the proteome of PfMig188. Table S2. The distribution of upregulated proteins across the different secretomes containing sugarcane bagasse. [file 13068_2021_2020_MOESM1_ESM.docx]

Additional file 1

for

Manuscript entitled “Accessory enzymes of *hypercellulolytic Penicillium funiculosum* facilitate complete saccharification of sugarcane bagasse”

Olusola A. Ogunyewo, Pooja Upadhyay, [Girish H. Rajachary](https://biotechnologyforbiofuels.biomedcentral.com/articles/10.1186/s13068-019-1516-6#auth-3)a, Omoaruemike E. Okereke, Laura Faas, Leonardo D. Gómez, Simon J. McQueen-Mason and Syed Shams Yazdani

**List of Items:**

**Table S1: The functional annotations of proteins identified in the proteome of *Pf*Mig1^88^**

**Table S2: The distribution of upregulated proteins across the different secretomes containing sugarcane bagasse**

**Table S1: The functional annotations of proteins identified in the proteome of *Pf*Mig1^88^**

| **Accession** | **Description** | **CAZY-Classification** | **PSMs** | **% coverage** | **Unique Peptides** | **AAs** | **MW [kDa]** | **calc. pI** | **Signal peptide** | **biological function** |  |
| --- | --- | --- | --- | --- | --- | --- | --- | --- | --- | --- | --- |
| maker-contig00012-exonerate_protein2genome-gene-4.113-mRNA-1 | cellobiohydrolase I | GH7-CBM1 | 1903 | 7.54 | 14 | 511 | 53.2 | 4.86 | yes | carbohydrate metabolic process |  |
| maker-contig00069-exonerate_protein2genome-gene-0.5-mRNA-1_1 | beta-glucosidase | GH3 | 1584 | 6.28 | 30 | 734 | 76.7 | 5.12 | yes | carbohydrate metabolic process |  |
| maker-contig00039-exonerate_protein2genome-gene-0.39-mRNA-1 | swollenin |  | 1245 | 4.93 | 16 | 332 | 35.7 | 4.87 | NO | carbohydrate binding |  |
| maker-contig00037-exonerate_protein2genome-gene-1.53-mRNA-1 | cellobiohydrolase II | GH6-CBM1 | 1014 | 4.02 | 11 | 464 | 48.4 | 4.91 | yes | carbohydrate metabolic process |  |
| maker-contig00012-exonerate_protein2genome-gene-2.40-mRNA-1 | Glucoamylase | GH15-CBM20 | 893 | 3.54 | 19 | 626 | 66.7 | 4.96 | yes | carbohydrate metabolic process |  |
| maker-contig00040-exonerate_protein2genome-gene-0.38-mRNA-1 | beta-glucosidase bgl3b | GH3 | 872 | 3.45 | 36 | 818 | 87.7 | 5 | yes | carbohydrate metabolic process |  |
| maker-contig00041-exonerate_protein2genome-gene-1.86-mRNA-1 | Endo-1,4-beta-xylanase D | GH10-CBM1 | 841 | 3.33 | 14 | 384 | 41 | 5.57 | yes | carbohydrate metabolic process |  |
| maker-contig00137-exonerate_protein2genome-gene-0.7-mRNA-1 | beta-xylosidase bxy3a | GH3 | 518 | 2.05 | 22 | 787 | 85.6 | 4.82 | yes | carbohydrate metabolic process |  |
| maker-contig00004-exonerate_protein2genome-gene-6.30-mRNA-1 | alpha-l-arabinofuranosidase | GH62 | 454 | 1.8 | 6 | 335 | 35.6 | 5.3 | yes | carbohydrate metabolic process |  |
| maker-contig00074-exonerate_protein2genome-gene-0.133-mRNA-1_1 | alpha-L-arabinofuranosidase | GH51-CBM42 | 452 | 1.79 | 13 | 465 | 48.5 | 5.44 | yes | carbohydrate metabolic process |  |
| maker-contig00013-exonerate_protein2genome-gene-3.69-mRNA-1 | hypothetical protein TCE0 | GH43-CBM6 | 392 | 1.55 | 12 | 410 | 44.5 | 4.96 | yes | carbohydrate metabolic process |  |
| maker-contig00005-exonerate_protein2genome-gene-2.76-mRNA-1 | alpha-l-arabinofuranosidase a | GH51 | 375 | 1.49 | 18 | 604 | 66.7 | 5 | yes | carbohydrate metabolic process |  |
| maker-contig00026-exonerate_protein2genome-gene-2.46-mRNA-1_1 | glycosyl hydrolase 5 protein | GH5 | 363 | 1.44 | 8 | 420 | 44.9 | 5.58 | yes | carbohydrate metabolic process |  |
| maker-contig00017-exonerate_protein2genome-gene-1.82-mRNA-1 | amidase family protein |  | 362 | 1.43 | 24 | 591 | 64.2 | 5.1 | yes | amino acid metabolism |  |
| maker-contig00012-exonerate_protein2genome-gene-4.76-mRNA-1 | acetic acid esterase | CE7-CBM1 | 336 | 1.33 | 10 | 263 | 28 | 5.17 | yes | carbohydrate metabolic process |  |
| maker-contig00012-exonerate_protein2genome-gene-2.13-mRNA-1 | hypothetical protein TCE0_039f13286 |  | 335 | 1.33 | 16 | 192 | 21.4 | 4.96 | NO | Other functions |  |
| maker-contig00020-exonerate_protein2genome-gene-2.23-mRNA-1 | choline dehydrogenase | AA3 | 327 | 1.3 | 14 | 545 | 57.9 | 4.54 | yes | carbohydrate metabolic process |  |
| maker-contig00013-exonerate_protein2genome-gene-3.62-mRNA-1 | hypothetical protein TCE0_015r02550 | GH27-CBM1 | 299 | 1.18 | 14 | 495 | 51.7 | 5.1 | yes | carbohydrate metabolic process |  |
| maker-contig00032-exonerate_protein2genome-gene-1.73-mRNA-1_1 | beta-glucosidase | GH1 | 290 | 1.15 | 19 | 609 | 68.5 | 4.94 | YES | carbohydrate metabolic process |  |
| maker-contig00084-exonerate_protein2genome-gene-0.91-mRNA-1 | Lactonase protein serp1457 |  | 286 | 1.13 | 11 | 394 | 41.7 | 4.69 | YES | Other functions |  |
| maker-contig00035-exonerate_protein2genome-gene-2.46-mRNA-1 | endo-beta-1,4 -glucanase cel5a | GH5-CBM1 | 281 | 1.11 | 5 | 395 | 42.7 | 4.59 | yes | carbohydrate metabolic process |  |
| maker-contig00106-exonerate_protein2genome-gene-0.28-mRNA-1_3 | GPI-anchored cell wall beta-1,3-endoglucanase | GH17 | 280 | 1.11 | 14 | 468 | 48.7 | 4.83 | YES | carbohydrate metabolic process |  |
| maker-contig00005-exonerate_protein2genome-gene-4.57-mRNA-1 | Glutaminase GtaA |  | 274 | 1.09 | 11 | 692 | 76.2 | 4.48 | YES | amino acid metabolism |  |
| maker-contig00094-exonerate_protein2genome-gene-0.18-mRNA-1_1 | Arabinogalactan endo-beta-1,4-galactanase | GH53 | 271 | 1.07 | 11 | 355 | 38.6 | 4.77 | YES | carbohydrate metabolic process |  |
| maker-contig00065-exonerate_protein2genome-gene-0.17-mRNA-1_1 | Alpha-1,2-mannosidase | GH92 | 270 | 1.07 | 20 | 804 | 88.1 | 5.11 | YES | carbohydrate metabolic process |  |
| maker-contig00030-exonerate_protein2genome-gene-0.45-mRNA-1 | pectate lyase | PL1 | 270 | 1.07 | 9 | 380 | 39.6 | 5.01 | yes | carbohydrate metabolic process |  |
| maker-contig00008-exonerate_protein2genome-gene-5.162-mRNA-1 | glycosyl hydrolase family 18 protein | GH13 | 246 | 0.97 | 7 | 313 | 33.9 | 4.61 | yes | carbohydrate metabolic process |  |
| maker-contig00012-exonerate_protein2genome-gene-2.63-mRNA-1 | glucan -alpha-glucosidase | GH15-CBM20 | 244 | 0.97 | 13 | 616 | 65.1 | 4.5 | yes | carbohydrate metabolic process |  |
| maker-contig00068-exonerate_protein2genome-gene-0.36-mRNA-1 | Alpha-L-arabinofuranosidase B | GH54-CBM42 | 238 | 0.94 | 10 | 465 | 48.5 | 4.79 | yes | carbohydrate metabolic process |  |
|  |  |  |  |  |  |  |  |  |  |  |  |
|  |  |  |  |  |  |  |  |  |  |  |  |
|  |  |  |  |  |  |  |  |  |  |  |  |
| maker-contig00094-exonerate_protein2genome-gene-0.70-mRNA-1 | mycelial catalase cat1 |  | 215 | 0.85 | 18 | 702 | 76.2 | 5.17 | YES | Oxidases with other functions |  |
| maker-contig00141-exonerate_protein2genome-gene-0.2-mRNA-1_1 | Immunoglobulin E- glycosyl hydrolase 5 | GH5 | 205 | 0.81 | 8 | 554 | 60.3 | 4.78 | yes | carbohydrate metabolic process |  |
| maker-contig00011-exonerate_protein2genome-gene-4.0-mRNA-1 | xyloglucanase cel74a | GH74-CBM1 | 203 | 0.8 | 15 | 842 | 88.4 | 5.08 | yes | carbohydrate metabolic process |  |
| maker-contig00045-exonerate_protein2genome-gene-1.133-mRNA-1 | isoamyl alcohol oxidase | AA7 | 201 | 0.8 | 12 | 565 | 60.6 | 4.88 | yes | carbohydrate metabolic process |  |
| maker-contig00104-exonerate_protein2genome-gene-0.23-mRNA-1 | extracellular glycosyl hydrolase | GH62-CBM1 | 199 | 0.79 | 7 | 388 | 41.2 | 4.96 | yes | carbohydrate metabolic process |  |
| maker-contig00054-exonerate_protein2genome-gene-1.59-mRNA-1 | rhamnogalacturonase a | GH28 | 196 | 0.78 | 9 | 434 | 45.9 | 5.45 | yes | carbohydrate metabolic process |  |
| maker-contig00062-exonerate_protein2genome-gene-0.106-mRNA-1_1 | Alpha-amylase | GH13-CBM20 | 190 | 0.75 | 8 | 610 | 66.6 | 4.51 | YES | carbohydrate metabolic process |  |
| maker-contig00004-exonerate_protein2genome-gene-7.100-mRNA-1 | alpha-glucosidase precursor | GH31 | 188 | 0.74 | 20 | 986 | 109 | 4.93 | yes | carbohydrate metabolic process |  |
| maker-contig00015-exonerate_protein2genome-gene-1.20-mRNA-1 | hypothetical protein TCE0_015f01617 | CE10 | 187 | 0.74 | 16 | 704 | 77 | 4.84 | yes | carbohydrate metabolic process |  |
| maker-contig00024-exonerate_protein2genome-gene-0.13-mRNA-1 | beta-galactosidase | GH3 | 186 | 0.74 | 18 | 728 | 80.2 | 5.26 | yes | carbohydrate metabolic process |  |
| maker-contig00042-exonerate_protein2genome-gene-1.32-mRNA-1_1 | beta-glucosidase | GH3 | 162 | 0.64 | 16 | 734 | 79.7 | 4.92 | yes | carbohydrate metabolic process |  |
| maker-contig00007-exonerate_protein2genome-gene-4.92-mRNA-1 | endo-beta-glucanase | GH12 | 159 | 0.63 | 5 | 236 | 25.5 | 5.81 | yes | carbohydrate metabolic process |  |
| maker-contig00005-exonerate_protein2genome-gene-2.92-mRNA-1 | swollenin |  | 156 | 0.62 | 6 | 291 | 31.4 | 4.41 | NO | carbohydrate binding |  |
| maker-contig00004-exonerate_protein2genome-gene-0.59-mRNA-1 | extracellular cell wall glucanase | GH16 | 142 | 0.56 | 6 | 411 | 41.5 | 4.54 | yes | carbohydrate metabolic process |  |
| maker-contig00005-exonerate_protein2genome-gene-3.32-mRNA-1 | cell wall - galactomannoprotein |  | 132 | 0.52 | 4 | 291 | 27.7 | 4.88 | YES | carbohydrate binding |  |
| maker-contig00047-exonerate_protein2genome-gene-0.53-mRNA-1 | acetyl xylan esterase | CE5-CBM1 | 132 | 0.52 | 2 | 283 | 28.4 | 4.87 | yes | carbohydrate metabolic process |  |
| maker-contig00002-exonerate_protein2genome-gene-5.32-mRNA-1 | endoglucanase esterase | CE2-CBM1 | 128 | 0.51 | 4 | 420 | 44.1 | 4.5 | yes | carbohydrate metabolic process |  |
| maker-contig00047-exonerate_protein2genome-gene-1.3-mRNA-1 | 1,3-beta-glucanosyltransferase | GH72 | 124 | 0.49 | 9 | 477 | 50.5 | 4.72 | yes | carbohydrate metabolic process |  |
| maker-contig00038-exonerate_protein2genome-gene-2.79-mRNA-1_1 | Alpha-glucosidase | GH31 | 119 | 0.47 | 14 | 894 | 99.1 | 4.81 | yes | carbohydrate metabolic process |  |
| maker-contig00004-exonerate_protein2genome-gene-7.52-mRNA-1 | alpha-amylase | GH13-CBM20 | 114 | 0.45 | 13 | 630 | 68.3 | 5.39 | yes | carbohydrate metabolic process |  |
| maker-contig00004-exonerate_protein2genome-gene-7.72-mRNA-1 | alpha-mannosidase family protein | GH92 | 111 | 0.44 | 12 | 882 | 96 | 4.86 | yes | carbohydrate metabolic process |  |
| maker-contig00018-exonerate_protein2genome-gene-3.7-mRNA-1 | hemolytic phospholipase c |  | 108 | 0.43 | 10 | 293 | 32.3 | 4.89 | yes | Other functions |  |
| maker-contig00222-exonerate_protein2genome-gene-0.3-mRNA-1_1 | Chitinase Glyco_18 domain-containing protein | GH18 | 107 | 0.42 | 11 | 914 | 98.5 | 4.36 | yes | carbohydrate metabolic process |  |
| maker-contig00048-exonerate_protein2genome-gene-1.96-mRNA-1 | cyanate hydratase |  | 106 | 0.42 | 10 | 163 | 18.2 | 6.58 | NO | Other functions |  |
| maker-contig00079-exonerate_protein2genome-gene-0.41-mRNA-1 | ubiquitin-40s ribosomal protein _ |  | 106 | 0.42 | 7 | 154 | 17.6 | 9.77 | NO | Other functions |  |
| maker-contig00004-exonerate_protein2genome-gene-6.48-mRNA-1 | glycosyl hydrolase family 61 protein | AA9 | 106 | 0.42 | 2 | 310 | 31.5 | 4.96 | yes | carbohydrate metabolic process |  |
| maker-contig00024-exonerate_protein2genome-gene-0.29-mRNA-1_1 | Glycosyl hydrolase | GH3 | 106 | 0.42 | 13 | 878 | 95 | 4.88 | Yes | carbohydrate metabolic process |  |
| maker-contig00109-exonerate_protein2genome-gene-0.47-mRNA-1_1 | Alpha-trehalose glucohydrolase TreA | GH65 | 106 | 0.42 | 11 | 1014 | 110 | 4.77 | yes | carbohydrate metabolic process |  |
| maker-contig00002-exonerate_protein2genome-gene-3.46-mRNA-1 | β-xylosidase | GH43 | 105 | 0.42 | 9 | 511 | 56.1 | 6.11 | yes | carbohydrate metabolic process |  |
| maker-contig00001-exonerate_protein2genome-gene-7.121-mRNA-1 | alpha-galactosidase | GH27 | 101 | 0.4 | 6 | 439 | 48.2 | 4.81 | yes | carbohydrate metabolic process |  |
| maker-contig00003-exonerate_protein2genome-gene-4.81-mRNA-1 | IgE-binding protein |  | 98 | 0.39 | 3 | 180 | 18.4 | 4.56 | yes | carbohydrate binding |  |
| maker-contig00025-exonerate_protein2genome-gene-2.66-mRNA-1 | allergen asp f 4 |  | 98 | 0.39 | 3 | 308 | 31.4 | 4.87 | Yes | Other functions |  |
| maker-contig00053-exonerate_protein2genome-gene-1.6-mRNA-1 | genome polyprotein | GH127 | 94 | 0.37 | 11 | 661 | 72.7 | 4.65 | yes | carbohydrate metabolic process |  |
| maker-contig00003-exonerate_protein2genome-gene-5.49-mRNA-1 | glycosyl hydrolase family 3 protein | GH3 | 94 | 0.37 | 8 | 780 | 84 | 5.15 | yes | carbohydrate metabolic process |  |
| maker-contig00005-exonerate_protein2genome-gene-2.10-mRNA-1_1 | Beta-hexosaminidase | GH20 | 93 | 0.37 | 6 | 603 | 66.8 | 4.98 | yes | carbohydrate metabolic process |  |
| maker-contig00015-exonerate_protein2genome-gene-3.38-mRNA-1 | glycoside hydrolase family 62 | GH62 | 89 | 0.35 | 6 | 332 | 35.3 | 4.79 | yes | carbohydrate metabolic process |  |
| maker-contig00154-exonerate_protein2genome-gene-0.0-mRNA-1 | carbohydrate-binding module family 1 protein | GH54-CBM1 | 88 | 0.35 | 8 | 399 | 40.7 | 5.36 | yes | carbohydrate metabolic process |  |
| maker-contig00012-exonerate_protein2genome-gene-2.25-mRNA-1 | alpha-amylase a type-3 | GH13 | 86 | 0.34 | 9 | 497 | 54.5 | 4.37 | yes | carbohydrate metabolic process |  |
| maker-contig00009-exonerate_protein2genome-gene-4.228-mRNA-1 | glucan endo- -alpha-glucosidase agn1 | GH71 | 86 | 0.34 | 9 | 435 | 46.9 | 4.46 | yes | carbohydrate metabolic process |  |
| maker-contig00022-exonerate_protein2genome-gene-3.24-mRNA-1 | aldose 1- epimerase |  | 84 | 0.33 | 5 | 402 | 43.3 | 4.73 | yes | carbohydrate binding |  |
| maker-contig00005-exonerate_protein2genome-gene-0.5-mRNA-1 | tannase and feruloyl esterase family protein |  | 80 | 0.32 | 7 | 556 | 60.5 | 5.12 | yes | carbohydrate binding |  |
| maker-contig00005-exonerate_protein2genome-gene-1.55-mRNA-1 | ferulic acid esterase A | CE1-CBM1 | 80 | 0.32 | 5 | 341 | 36 | 6.1 | yes | carbohydrate metabolic process |  |
| maker-contig00113-exonerate_protein2genome-gene-0.52-mRNA-1_1 | uncharacterized protein |  | 80 | 0.32 | 10 | 641 | 68.9 | 4.91 | Yes | Other functions |  |
| maker-contig00012-exonerate_protein2genome-gene-2.58-mRNA-1 | transcription factor - _--_ |  | 79 | 0.31 | 12 | 417 | 44.8 | 5.36 | NO | Other functions |  |
| P17493 | Bleomycin resistance protein |  | 79 | 0.31 | 4 | 124 | 13.8 | 4.3 | NO | Other functions |  |
| maker-contig00005-exonerate_protein2genome-gene-6.188-mRNA-1 | xylan -beta-xylosidase | GH3 | 75 | 0.3 | 11 | 738 | 79.5 | 4.84 | yes | carbohydrate metabolic process |  |
| maker-contig00017-exonerate_protein2genome-gene-2.95-mRNA-1_1 | Beta-glucosidase GH3 | GH3 | 74 | 0.29 | 10 | 591 | 63.6 | 4.56 | yes | carbohydrate metabolic process |  |
| maker-contig00035-exonerate_protein2genome-gene-2.66-mRNA-1 | wsc domain containing protein | AA5 | 72 | 0.29 | 11 | 1046 | 111.2 | 4.48 | yes | carbohydrate metabolic process |  |
| maker-contig00094-exonerate_protein2genome-gene-0.81-mRNA-1 | beta-glucosidase | GH3 | 72 | 0.29 | 12 | 723 | 77.5 | 4.83 | yes | carbohydrate metabolic process |  |
| maker-contig00020-exonerate_protein2genome-gene-1.98-mRNA-1 | hypothetical protein | CE10 | 70 | 0.28 | 12 | 509 | 55.9 | 5.07 | yes | carbohydrate metabolic process |  |
| maker-contig00059-exonerate_protein2genome-gene-1.55-mRNA-1 | Dextranase | GH49 | 66 | 0.26 | 9 | 605 | 66 | 5.07 | yes | carbohydrate metabolic process |  |
| maker-contig00011-exonerate_protein2genome-gene-3.42-mRNA-1 | multidrug transporter |  | 66 | 0.26 | 3 | 367 | 39.1 | 4.84 | yes | Other functions |  |
| maker-contig00021-exonerate_protein2genome-gene-2.102-mRNA-1 | hydrophobic surface binding protein A |  | 65 | 0.26 | 5 | 173 | 17.7 | 4.12 |  | carbohydrate binding |  |
| maker-contig00012-exonerate_protein2genome-gene-1.39-mRNA-1 | alpha-galactosidase D | GH27 | 64 | 0.25 | 8 | 611 | 65.6 | 4.78 | yes | carbohydrate metabolic process |  |
| P02858 | Glycinin |  | 61 | 0.24 | 3 | 563 | 63.8 | 5.29 | YES | carbohydrate binding |  |
| maker-contig00014-exonerate_protein2genome-gene-1.10-mRNA-1 | hypothetical protein TCE0_017f04250 - _--_ |  | 60 | 0.24 | 5 | 198 | 22.3 | 5.83 | NO | Other functions |  |
| maker-contig00105-exonerate_protein2genome-gene-0.26-mRNA-1 | isoamyl alcohol oxidase | AA7 | 60 | 0.24 | 5 | 611 | 65.2 | 4.93 | yes | carbohydrate metabolic process |  |
| maker-contig00006-exonerate_protein2genome-gene-4.70-mRNA-1 | aminopeptidase 2 |  | 58 | 0.23 | 20 | 889 | 99.2 | 5.44 | NO | Amino acid metabolism |  |
| maker-contig00001-exonerate_protein2genome-gene-1.101-mRNA-1 | autophagic serine protease alp2 |  | 56 | 0.22 | 6 | 490 | 51.9 | 6.1 | NO | amino acid metabolism |  |
| maker-contig00048-exonerate_protein2genome-gene-1.122-mRNA-1_1 | Feruloyl esterase B |  | 53 | 0.21 | 3 | 323 | 34.2 | 5.91 | Yes | carbohydrate binding |  |
| maker-contig00083-exonerate_protein2genome-gene-0.12-mRNA-1 | Neutral ceramidase |  | 52 | 0.21 | 9 | 753 | 81.2 | 4.83 | yes | Other functions |  |
| maker-contig00043-exonerate_protein2genome-gene-1.174-mRNA-1 | beta-1,3-glucanosyltransferase | GH72-CBM43 | 49 | 0.19 | 3 | 517 | 54.6 | 4.51 | yes | carbohydrate metabolic process |  |
| maker-contig00024-exonerate_protein2genome-gene-3.92-mRNA-1 | glucan -beta-glucosidase | GH55 | 48 | 0.19 | 5 | 792 | 83.2 | 4.73 | yes | carbohydrate metabolic process |  |
| maker-contig00078-exonerate_protein2genome-gene-0.30-mRNA-1_1 | Putative glycosyl hydrolase family 16 | GH16 | 47 | 0.19 | 6 | 286 | 30.8 | 4.87 | yes | carbohydrate metabolic process |  |
| maker-contig00010-exonerate_protein2genome-gene-2.14-mRNA-1 | endo-beta- 1,4-galactanase | GH30 | 47 | 0.19 | 8 | 487 | 53.4 | 5.39 | yes | carbohydrate metabolic process |  |
| maker-contig00025-exonerate_protein2genome-gene-1.64-mRNA-1 | extracellular | GH71-CBM24 | 47 | 0.19 | 7 | 551 | 58.3 | 4.75 | yes | carbohydrate metabolic process |  |
| maker-contig00001-exonerate_protein2genome-gene-8.0-mRNA-1 | hypothetical protein TCE0 |  | 47 | 0.19 | 4 | 302 | 33.1 | 4.55 | Yes | Other functions |  |
| maker-contig00063-exonerate_protein2genome-gene-0.135-mRNA-1 | 1,3-beta-glucanosyltransferase | GH72 | 46 | 0.18 | 5 | 447 | 47.9 | 4.91 | yes | carbohydrate metabolic process |  |
| maker-contig00035-exonerate_protein2genome-gene-2.65-mRNA-1 | fungistatic metabolite | AA2 | 45 | 0.18 | 6 | 399 | 42.6 | 4.65 | yes | carbohydrate metabolic process |  |
| maker-contig00011-exonerate_protein2genome-gene-1.103-mRNA-1 | lipase 1 | CE10 | 45 | 0.18 | 6 | 520 | 56.3 | 4.78 | yes | carbohydrate metabolic process |  |
| maker-contig00132-exonerate_protein2genome-gene-0.8-mRNA-1 | endo-1,4-beta- xylanase | GH11-CBM1 | 45 | 0.18 | 2 | 282 | 29.5 | 5.15 | yes | carbohydrate metabolic process |  |
| maker-contig00061-exonerate_protein2genome-gene-1.39-mRNA-1 | bnr asp-box repeat domain protein | GH93 | 45 | 0.18 | 9 | 376 | 41.1 | 5.2 | yes | carbohydrate metabolic process |  |
| maker-contig00035-exonerate_protein2genome-gene-1.82-mRNA-1 | bifunctional catalase-peroxidase cat2 | AA2 | 43 | 0.17 | 13 | 733 | 80.7 | 6.32 | yes | carbohydrate metabolic process |  |
| maker-contig00068-exonerate_protein2genome-gene-0.63-mRNA-1 | alpha beta-glucosidase agdc | GH31 | 41 | 0.16 | 8 | 926 | 104.1 | 4.93 | yes | carbohydrate metabolic process |  |
| maker-contig00055-exonerate_protein2genome-gene-1.29-mRNA-1 | hypothetical protein TCE0 |  | 41 | 0.16 | 3 | 104 | 11.2 | 4.65 | Yes | Oxidases with other functions |  |
| maker-contig00031-exonerate_protein2genome-gene-1.39-mRNA-1 | alanine-glyoxylate transaminase |  | 39 | 0.15 | 7 | 386 | 41.3 | 6.67 | NO | Amino acid metabolism |  |
| maker-contig00062-exonerate_protein2genome-gene-0.34-mRNA-1 | FAD dependent oxidoreductase | AA7 | 39 | 0.15 | 4 | 498 | 54.4 | 4.36 | yes | carbohydrate metabolic process |  |
| maker-contig00015-exonerate_protein2genome-gene-4.29-mRNA-1 | hsp70 chaperone |  | 39 | 0.15 | 6 | 673 | 73.5 | 5 | yes | Other functions |  |
| maker-contig00013-exonerate_protein2genome-gene-1.73-mRNA-1_1 | Lipase_GDSL domain-containing protein |  | 38 | 0.15 | 3 | 359 | 40.1 | 4.86 | YES | Lipid metabolism |  |
| maker-contig00070-exonerate_protein2genome-gene-0.125-mRNA-1_1 | FAD-binding PCMH-type domain-containing protein |  | 37 | 0.15 | 5 | 419 | 45 | 4.69 | Yes | Oxidases with other functions |  |
| maker-contig00017-exonerate_protein2genome-gene-0.8-mRNA-1 | carboxypeptidase y like A |  | 35 | 0.14 | 6 | 551 | 61.6 | 4.88 | yes | amino acid metabolism |  |
| maker-contig00028-exonerate_protein2genome-gene-2.37-mRNA-1 | Cytochrome c domain-containing protein |  | 34 | 0.13 | 2 | 103 | 11.3 | 9.03 | Yes | Oxidases with other functions |  |
| maker-contig00027-exonerate_protein2genome-gene-2.48-mRNA-1_1 | Peptidase_S9 domain-containing protein |  | 33 | 0.13 | 5 | 727 | 80 | 5.49 | NO | Amino acid metabolism |  |
| maker-contig00011-exonerate_protein2genome-gene-4.30-mRNA-1 | Endo-1,4-beta-xylanase | GH11 | 33 | 0.13 | 1 | 155 | 16.8 | 5.21 | yes | carbohydrate metabolic process |  |
| maker-contig00026-exonerate_protein2genome-gene-2.30-mRNA-1 | Extracellular exo-polygalacturonase | GH28 | 33 | 0.13 | 4 | 434 | 47 | 5.22 | yes | carbohydrate metabolic process |  |
| maker-contig00022-exonerate_protein2genome-gene-0.7-mRNA-1_1 | Protein kinase domain-containing protein |  | 32 | 0.13 | 5 | 158 | 18.8 | 6.24 | NO | Other functions |  |
| maker-contig00005-exonerate_protein2genome-gene-4.25-mRNA-1 | alkaline serine protease |  | 32 | 0.13 | 3 | 648 | 69.3 | 4.63 | yes | amino acid metabolism |  |
| maker-contig00108-exonerate_protein2genome-gene-0.76-mRNA-1 | rhamnogalacturonan acetylesterase | CE12 | 32 | 0.13 | 3 | 255 | 26.8 | 5.2 | yes | carbohydrate metabolic process |  |
| maker-contig00042-exonerate_protein2genome-gene-1.114-mRNA-1 | xyloglucan-specific endo-beta-1,4-glucanase | GH12 | 32 | 0.13 | 2 | 246 | 26.3 | 4.25 | yes | carbohydrate metabolic process |  |
| maker-contig00010-exonerate_protein2genome-gene-1.9-mRNA-1 | fructose-bisphosphate aldolase |  | 31 | 0.12 | 10 | 362 | 39.9 | 5.69 | NO | carbohydrate binding |  |
| maker-contig00039-exonerate_protein2genome-gene-2.44-mRNA-1_1 | glucan 1,4-alpha-glucosidase | GH31 | 31 | 0.12 | 5 | 921 | 101.3 | 5.01 | YES | carbohydrate metabolic process |  |
| maker-contig00194-exonerate_protein2genome-gene-0.12-mRNA-1_1 | Uncharacterized protein |  | 30 | 0.12 | 2 | 593 | 64.9 | 4.75 | NO | Oxidases with other functions |  |
| maker-contig00037-exonerate_protein2genome-gene-1.51-mRNA-1 | polysaccharide lyase family 1 protein | PL1 | 30 | 0.12 | 4 | 360 | 37.5 | 4.92 | yes | carbohydrate metabolic process |  |
| maker-contig00014-exonerate_protein2genome-gene-0.47-mRNA-1_1 | GPI anchored protein |  | 30 | 0.12 | 3 | 366 | 40.5 | 5.87 | yes | Other functions |  |
| P02769 | Serum albumin |  | 29 | 0.11 | 7 | 607 | 69.2 | 6.18 | NO | Other functions |  |
| maker-contig00055-exonerate_protein2genome-gene-1.59-mRNA-1 | mucin signaling protein |  | 29 | 0.11 | 2 | 674 | 67 | 5.02 | NO | Other functions |  |
| maker-contig00003-exonerate_protein2genome-gene-5.44-mRNA-1 | exoinulinase | GH32 | 29 | 0.11 | 6 | 505 | 55.5 | 5.02 | yes | carbohydrate metabolic process |  |
| maker-contig00037-exonerate_protein2genome-gene-1.73-mRNA-1 | mannan endo- -alpha-mannosidase dcw1 | GH76 | 29 | 0.11 | 4 | 459 | 50.1 | 4.58 | yes | carbohydrate metabolic process |  |
| maker-contig00009-exonerate_protein2genome-gene-4.160-mRNA-1 | xaa-pro aminopeptidase pepp |  | 28 | 0.11 | 8 | 468 | 52.1 | 5.55 | No | amino acid metabolism |  |
| maker-contig00066-exonerate_protein2genome-gene-0.3-mRNA-1_1 | Endo-1,4-beta-mannosidase | GH5 | 28 | 0.11 | 2 | 441 | 46.6 | 4.64 | yes | carbohydrate metabolic process |  |
| maker-contig00052-exonerate_protein2genome-gene-1.137-mRNA-1_2 | S-adenosyl-L-homocysteine hydrolase |  | 27 | 0.11 | 5 | 468 | 51 | 6.52 | NO | Amino acid metabolism |  |
| maker-contig00055-exonerate_protein2genome-gene-1.5-mRNA-1_1 | Phenazine biosynthesis-like protein |  | 27 | 0.11 | 5 | 326 | 34.9 | 5.5 | NO | Other functions |  |
| maker-contig00006-exonerate_protein2genome-gene-5.81-mRNA-1 | cell wall glucanase | GH16-CBM18 | 26 | 0.1 | 2 | 436 | 45.7 | 4.35 | yes | carbohydrate metabolic process |  |
| maker-contig00024-exonerate_protein2genome-gene-0.17-mRNA-1 | probable beta-galactosidase | GH35 | 26 | 0.1 | 3 | 202 | 22.1 | 4.84 | yes | carbohydrate metabolic process |  |
| maker-contig00025-exonerate_protein2genome-gene-0.32-mRNA-1_1 | 6,7-dimethyl-8-ribityllumazine synthase |  | 25 | 0.1 | 5 | 205 | 21.7 | 6.42 | NO | Other functions |  |
| maker-contig00055-exonerate_protein2genome-gene-0.79-mRNA-1 | Carboxylic ester hydrolase | CE10 | 25 | 0.1 | 2 | 176 | 18.4 | 4.48 | yes | carbohydrate metabolic process |  |
| maker-contig00082-exonerate_protein2genome-gene-0.106-mRNA-1 | cutinase | CE5 | 25 | 0.1 | 3 | 252 | 26.4 | 4.44 | yes | carbohydrate metabolic process |  |
| maker-contig00048-exonerate_protein2genome-gene-1.138-mRNA-1 | endo- 1,4-beta-xylanase | GH11-CBM1 | 25 | 0.1 | 2 | 276 | 29.2 | 4.96 | yes | carbohydrate metabolic process |  |
| maker-contig00001-exonerate_protein2genome-gene-11.72-mRNA-1 | beta-glucosidase | GH3 | 24 | 0.1 | 11 | 829 | 90.8 | 5.71 | yes | carbohydrate metabolic process |  |
| maker-contig00002-exonerate_protein2genome-gene-4.51-mRNA-1 | glycoside hydrolase family 5 protein | GH30 | 24 | 0.1 | 4 | 462 | 51 | 5.05 | yes | carbohydrate metabolic process |  |
| maker-contig00003-exonerate_protein2genome-gene-5.3-mRNA-1 | Putative β-xylosidase | GH43 | 24 | 0.1 | 4 | 519 | 57.1 | 5.26 | yes | carbohydrate metabolic process |  |
| maker-contig00006-exonerate_protein2genome-gene-2.24-mRNA-1 | lysophospholipase plb1 |  | 23 | 0.09 | 3 | 632 | 67.4 | 4.59 | NO | Lipid metabolism |  |
| Q9HFH0 | Endo-1,4-beta-xylanase | GH11 | 23 | 0.09 | 2 | 223 | 24 | 4.6 | Yes | carbohydrate metabolic process |  |
| maker-contig00019-exonerate_protein2genome-gene-0.23-mRNA-1_1 | Galactan 1,3-beta-galactosidase | GH43 | 22 | 0.09 | 3 | 451 | 48.6 | 4.87 | yes | carbohydrate metabolic process |  |
| maker-contig00016-exonerate_protein2genome-gene-3.167-mRNA-1 | mannosidase | GH47 | 22 | 0.09 | 3 | 502 | 55.1 | 4.58 | yes | carbohydrate metabolic process |  |
| maker-contig00001-exonerate_protein2genome-gene-5.82-mRNA-1 | aspartic endopeptidase |  | 21 | 0.08 | 1 | 340 | 36.1 | 5.21 | YES | Amino acid metabolism |  |
| maker-contig00048-exonerate_protein2genome-gene-1.66-mRNA-1 | duf1237 domain protein | GH125 | 21 | 0.08 | 2 | 907 | 100.1 | 5.06 | yes | carbohydrate metabolic process |  |
| maker-contig00016-exonerate_protein2genome-gene-2.47-mRNA-1 | beta-galactosidase | GH35 | 21 | 0.08 | 1 | 1010 | 109.3 | 5.16 | yes | carbohydrate metabolic process |  |
| maker-contig00003-exonerate_protein2genome-gene-5.0-mRNA-1_1 | Putative Beta-xylosidase | GH5 | 21 | 0.08 | 3 | 459 | 51.2 | 5.16 | YES | carbohydrate metabolic process |  |
| maker-contig00061-exonerate_protein2genome-gene-0.34-mRNA-1 | envelope glycoprotein | GH53-CBM61 | 21 | 0.08 | 2 | 563 | 56.6 | 8.44 | yes | carbohydrate metabolic process |  |
| maker-contig00064-exonerate_protein2genome-gene-0.26-mRNA-1 | endo-beta-1,4-glucanase cel7b | GH7-CBM1 | 21 | 0.08 | 3 | 461 | 47.7 | 4.7 | yes | carbohydrate metabolic process |  |
| P0DO16 | Beta-conglycinin |  | 21 | 0.08 | 3 | 605 | 70.3 | 5.17 | Yes | Other functions |  |
| maker-contig00104-exonerate_protein2genome-gene-0.20-mRNA-1 | endo-β-1,4-xylanase | GH30-CBM1 | 20 | 0.08 | 6 | 524 | 54.8 | 4.77 | yes | carbohydrate metabolic process |  |
| maker-contig00019-exonerate_protein2genome-gene-1.69-mRNA-1 | glycoside hydrolase family 79 protein | GH79 | 20 | 0.08 | 2 | 476 | 49.7 | 5.39 | yes | carbohydrate metabolic process |  |
| maker-contig00044-exonerate_protein2genome-gene-0.89-mRNA-1_1 | Peptidase S10, serine carboxypeptidase |  | 19 | 0.08 | 4 | 640 | 70.7 | 4.49 | Yes | Amino acid metabolism |  |
| maker-contig00046-exonerate_protein2genome-gene-1.128-mRNA-1 | hypothetical protein TCE0_044r17570 |  | 19 | 0.08 | 2 | 190 | 19.2 | 4.56 | Yes | Other functions |  |
| maker-contig00022-exonerate_protein2genome-gene-2.37-mRNA-1 | aspartic endopeptidase pep2 EC-3.4.23 |  | 18 | 0.07 | 4 | 395 | 42.8 | 4.86 | NO | Amino acid metabolism |  |
| maker-contig00088-exonerate_protein2genome-gene-0.122-mRNA-1 | Extracellular serine carboxypeptidase |  | 18 | 0.07 | 3 | 561 | 63 | 4.74 | Yes | amino acid metabolism |  |
| maker-contig00037-exonerate_protein2genome-gene-0.55-mRNA-1 | class v Chitinase | GH18 | 18 | 0.07 | 4 | 386 | 42 | 4.96 | yes | carbohydrate metabolic process |  |
| maker-contig00068-exonerate_protein2genome-gene-0.8-mRNA-1 | hypothetical protein TCE0_011f00638 | GH93 | 18 | 0.07 | 4 | 355 | 37.1 | 4.75 | yes | carbohydrate metabolic process |  |
| maker-contig00022-exonerate_protein2genome-gene-2.75-mRNA-1_1 | Transthyretin domain protein |  | 17 | 0.07 | 2 | 174 | 19.7 | 6.8 | NO | Other functions |  |
| maker-contig00055-exonerate_protein2genome-gene-1.102-mRNA-1 | non-hemolytic phospholipase c |  | 17 | 0.07 | 4 | 617 | 68.4 | 4.83 | Yes | Lipid metabolism |  |
| maker-contig00142-exonerate_protein2genome-gene-0.49-mRNA-1_1 | Histidine phosphatase superfamily |  | 16 | 0.06 | 2 | 510 | 55.1 | 4.96 | NO | amino acid metabolism |  |
| maker-contig00017-exonerate_protein2genome-gene-2.5-mRNA-1 | sphingomyelin phosphodiesterase b |  | 16 | 0.06 | 2 | 627 | 67.8 | 4.72 | NO | Lipid metabolism |  |
| maker-contig00011-exonerate_protein2genome-gene-2.48-mRNA-1 | allergenic cerato-platanin asp f13 |  | 16 | 0.06 | 1 | 162 | 16.6 | 4.35 | No | Other functions |  |
| maker-contig00013-exonerate_protein2genome-gene-3.7-mRNA-1 | ribose 5-phosphate isomerase a |  | 16 | 0.06 | 3 | 320 | 34.2 | 9.17 | NO | Other functions |  |
| maker-contig00062-exonerate_est2genome-gene-0.2-mRNA-1_1 | NmrA domain-containing protein |  | 16 | 0.06 | 2 | 213 | 23.9 | 4.77 | No | Other functions |  |
| maker-contig00103-exonerate_protein2genome-gene-0.20-mRNA-1 | Peptide hydrolase |  | 16 | 0.06 | 3 | 368 | 39.4 | 4.65 | Yes | Amino acid metabolism |  |
| maker-contig00087-exonerate_protein2genome-gene-0.10-mRNA-1 | pectinesterase a | CE8 | 16 | 0.06 | 2 | 404 | 43.7 | 5.8 | yes | carbohydrate metabolic process |  |
| maker-contig00067-exonerate_protein2genome-gene-0.93-mRNA-1 | lactoylglutathione lyase |  | 16 | 0.06 | 2 | 377 | 41.4 | 5.24 | Yes | Other functions |  |
| maker-contig00080-exonerate_protein2genome-gene-0.55-mRNA-1 | 3-isopropylmalate dehydrogenase leu2a |  | 15 | 0.06 | 4 | 365 | 38.9 | 5.58 | NO | Oxidases with other functions |  |
| maker-contig00116-exonerate_protein2genome-gene-0.6-mRNA-1 | beta-fructofuranosidase | GH32 | 15 | 0.06 | 2 | 482 | 53.3 | 5.2 | yes | carbohydrate metabolic process |  |
| maker-contig00093-exonerate_protein2genome-gene-0.51-mRNA-1 | Exo-beta-1,3-glucanase | GH55 | 15 | 0.06 | 3 | 788 | 82.2 | 4.56 | yes | carbohydrate metabolic process |  |
| maker-contig00141-exonerate_protein2genome-gene-0.28-mRNA-1 | Gpi anchored dioxygenase |  | 15 | 0.06 | 3 | 376 | 40.8 | 4.79 | YES | Oxidases with other functions |  |
| maker-contig00001-exonerate_protein2genome-gene-1.49-mRNA-1 | Superoxide dismutase |  | 14 | 0.06 | 3 | 1038 | 114 | 6.47 | NO | Oxidases with other functions |  |
| maker-contig00010-exonerate_protein2genome-gene-2.36-mRNA-1 | fad-dependent oxidase | AA7 | 14 | 0.06 | 3 | 404 | 43.1 | 4.64 | yes | carbohydrate metabolic process |  |
| maker-contig00022-exonerate_protein2genome-gene-3.22-mRNA-1 | beta-1,3-glucanosyltransferase | GH72 | 14 | 0.06 | 1 | 437 | 45.8 | 4.7 | yes | carbohydrate metabolic process |  |
| maker-contig00051-exonerate_protein2genome-gene-1.88-mRNA-1 | glucan |  | 14 | 0.06 | 2 | 228 | 22.5 | 4.96 | YES | Lipid metabolism |  |
| maker-contig00001-exonerate_protein2genome-gene-7.110-mRNA-1 | hypothetical protein TCE0_018f05417 |  | 14 | 0.06 | 2 | 255 | 26.9 | 5.08 | Yes | Other functions |  |
| maker-contig00013-exonerate_protein2genome-gene-1.72-mRNA-1 | hypothetical protein TCE0_015r02424 |  | 14 | 0.06 | 1 | 83 | 8.7 | 4.53 | yes | Other functions |  |
| maker-contig00061-exonerate_protein2genome-gene-0.50-mRNA-1 | amidohydrolase family protein |  | 13 | 0.05 | 6 | 319 | 36.9 | 6.09 | NO | amino acid metabolism |  |
| P41757 | Phosphoglycerate kinase |  | 13 | 0.05 | 1 | 417 | 44.9 | 6.05 | NO | Other functions |  |
| maker-contig00051-exonerate_protein2genome-gene-0.77-mRNA-1 | para-nitrobenzyl esterase | CE10 | 13 | 0.05 | 3 | 454 | 50.3 | 4.56 | yes | carbohydrate metabolic process |  |
| maker-contig00124-exonerate_protein2genome-gene-0.48-mRNA-1 | Beta-galactosidase | GH35 | 13 | 0.05 | 4 | 936 | 103.2 | 5.03 | yes | carbohydrate metabolic process |  |
| maker-contig00020-exonerate_protein2genome-gene-3.18-mRNA-1 | phosphatidylglycerol specific phospholipase |  | 12 | 0.05 | 2 | 470 | 51.4 | 5.05 | No | Lipid metabolism |  |
| maker-contig00007-exonerate_protein2genome-gene-4.121-mRNA-1 | DlpA domain protein |  | 12 | 0.05 | 3 | 288 | 30.8 | 8.97 | NO | Other functions |  |
| maker-contig00016-exonerate_protein2genome-gene-2.7-mRNA-1 | sun domain protein | GH132 | 12 | 0.05 | 2 | 458 | 46.1 | 5.01 | yes | carbohydrate metabolic process |  |
| maker-contig00151-exonerate_protein2genome-gene-0.2-mRNA-1_1 | Carboxylic ester hydrolase |  | 12 | 0.05 | 3 | 371 | 40.1 | 4.77 | yes | Other functions |  |
| maker-contig00034-exonerate_protein2genome-gene-2.61-mRNA-1 | Allantoicase |  | 12 | 0.05 | 3 | 370 | 40.2 | 6.29 | Yes | Other functions |  |
| maker-contig00115-exonerate_protein2genome-gene-0.0-mRNA-1 | aspartic-type endopeptidase opsb |  | 11 | 0.04 | 3 | 469 | 48.4 | 4.72 | NO | amino acid metabolism |  |
| maker-contig00001-exonerate_protein2genome-gene-6.92-mRNA-1 | Nucleosome assembly protein (NAP) family protein |  | 11 | 0.04 | 4 | 367 | 41.8 | 4.31 | No | Other functions |  |
| maker-contig00008-exonerate_protein2genome-gene-3.1-mRNA-1 | histidine phosphatase superfamily clade-2 |  | 11 | 0.04 | 1 | 540 | 59.5 | 4.86 | Yes | amino acid metabolism |  |
| maker-contig00104-exonerate_protein2genome-gene-0.42-mRNA-1 | GPI anchored CFEM domain protein C |  | 11 | 0.04 | 1 | 178 | 16.5 | 6.86 | Yes | carbohydrate binding |  |
| maker-contig00005-exonerate_protein2genome-gene-3.16-mRNA-1 | Exo-beta-1,3-glucanase | GH55 | 11 | 0.04 | 3 | 579 | 60.2 | 4.91 | yes | carbohydrate metabolic process |  |
| maker-contig00097-exonerate_protein2genome-gene-0.15-mRNA-1 | beta-lactamase, putative |  | 11 | 0.04 | 3 | 442 | 48.5 | 5.3 | yes | Other functions |  |
| maker-contig00063-exonerate_protein2genome-gene-0.50-mRNA-1 | fumarylacetoacetate hydrolase family protein |  | 10 | 0.04 | 4 | 303 | 32.9 | 5.44 | No | Other functions |  |
| maker-contig00070-exonerate_protein2genome-gene-0.119-mRNA-1_1 | GMC oxidoreductase |  | 10 | 0.04 | 3 | 623 | 67.6 | 5.35 | NO | Oxidases with other functions |  |
| maker-contig00038-exonerate_protein2genome-gene-2.76-mRNA-1_1 | Carboxypeptidase |  | 10 | 0.04 | 2 | 517 | 56.8 | 4.51 | YES | Amino acid metabolism |  |
| maker-contig00105-exonerate_protein2genome-gene-0.59-mRNA-1 | fad-binding oxidoreductase | AA7 | 10 | 0.04 | 1 | 471 | 50.1 | 5.07 | yes | carbohydrate metabolic process |  |
| maker-contig00140-exonerate_protein2genome-gene-0.2-mRNA-1 | Endo-1,4-beta-xylanase | GH11 | 10 | 0.04 | 1 | 211 | 22.7 | 4.64 | yes | carbohydrate metabolic process |  |
| maker-contig00034-exonerate_protein2genome-gene-0.23-mRNA-1 | [GPI anchored endo-1,3(4)-beta-glucanase](https://blast.ncbi.nlm.nih.gov/Blast.cgi#alnHdr_GAM41795) | GH16 | 10 | 0.04 | 1 | 546 | 54.9 | 4.96 | yes | carbohydrate metabolic process |  |
| maker-contig00074-exonerate_protein2genome-gene-0.108-mRNA-1 | allergen asp f7 - _--_ |  | 10 | 0.04 | 1 | 242 | 24.1 | 4.32 | Yes | Other functions |  |
| maker-contig00001-exonerate_protein2genome-gene-11.151-mRNA-1 | nucleoside diphosphate kinase |  | 9 | 0.04 | 3 | 153 | 16.9 | 8.12 | No | Other functions |  |
| maker-contig00001-exonerate_protein2genome-gene-11.202-mRNA-1 | aldehyde reductase |  | 9 | 0.04 | 5 | 336 | 37.1 | 5.96 | NO | Oxidases with other functions |  |
| Q02257 | Junction plakoglobin |  | 8 | 0.03 | 5 | 745 | 81.7 | 6.14 | NO | Other functions |  |
| maker-contig00036-exonerate_protein2genome-gene-2.103-mRNA-1_1 | SMC hinge domain-containing protein |  | 8 | 0.03 | 1 | 1180 | 132.8 | 6.99 | NO | Other functions |  |
| maker-contig00056-exonerate_protein2genome-gene-1.100-mRNA-1 | reticuline oxidase-like protein | AA7 | 8 | 0.03 | 2 | 474 | 50.9 | 4.44 | yes | carbohydrate metabolic process |  |
| maker-contig00022-exonerate_protein2genome-gene-0.79-mRNA-1 | Endo-1,4-beta-xylanase | GH11 | 8 | 0.03 | 1 | 256 | 27.4 | 5.83 | yes | carbohydrate metabolic process |  |
| maker-contig00014-exonerate_protein2genome-gene-2.42-mRNA-1_1 | Beta_hexosaminidase | GH16 | 8 | 0.03 | 2 | 373 | 39.5 | 4.56 | yes | carbohydrate metabolic process |  |
| maker-contig00003-exonerate_protein2genome-gene-2.17-mRNA-1 | Glycosyl hydrolase | GH2 | 8 | 0.03 | 2 | 892 | 100 | 4.93 | yes | carbohydrate metabolic process |  |
| maker-contig00024-exonerate_protein2genome-gene-1.36-mRNA-1 | beta-d-glucuronoside glucuronosohydrolase flags | GH79 | 8 | 0.03 | 2 | 526 | 57.4 | 5.48 | yes | carbohydrate metabolic process |  |
| maker-contig00085-exonerate_protein2genome-gene-0.76-mRNA-1 | ML domain-containing protein |  | 8 | 0.03 | 1 | 169 | 18.6 | 4.59 | Yes | Lipid metabolism |  |
| maker-contig00018-exonerate_protein2genome-gene-3.53-mRNA-1_1 | Purple acid phosphatase |  | 8 | 0.03 | 1 | 493 | 53.3 | 4.88 | yes | Other functions |  |
| maker-contig00074-exonerate_protein2genome-gene-0.145-mRNA-1 | gpi-anchored cell wall organization protein ecm33 - _--_ |  | 8 | 0.03 | 1 | 398 | 40.4 | 4.3 | Yes | Other functions |  |
| maker-contig00016-exonerate_protein2genome-gene-3.222-mRNA-1 | aminoacylproline aminopeptidase |  | 7 | 0.03 | 3 | 307 | 33.3 | 5.06 | NO | Amino acid metabolism |  |
| maker-contig00146-exonerate_protein2genome-gene-0.18-mRNA-1_1 | Mitochondrial glycoprotein |  | 7 | 0.03 | 3 | 296 | 33.4 | 4.65 | NO | Other functions |  |
| maker-contig00001-exonerate_protein2genome-gene-1.31-mRNA-1_3 | Transaldolase |  | 7 | 0.03 | 4 | 342 | 37.3 | 6.46 | NO | Other functions |  |
| maker-contig00018-exonerate_protein2genome-gene-2.30-mRNA-1 | Carbohydrate-binding domain, family 9-like |  | 7 | 0.03 | 1 | 227 | 25.3 | 4.97 | Yes | carbohydrate binding |  |
| maker-contig00013-exonerate_protein2genome-gene-0.40-mRNA-1 | cellulase family protein | GH5 | 7 | 0.03 | 3 | 409 | 45.2 | 4.82 | yes | carbohydrate metabolic process |  |
| maker-contig00008-exonerate_protein2genome-gene-5.150-mRNA-1 | glucan endo- -alpha-glucosidase agn1 | GH71 | 7 | 0.03 | 1 | 390 | 42.8 | 4.4 | yes | carbohydrate metabolic process |  |
| maker-contig00043-exonerate_protein2genome-gene-0.56-mRNA-1 | conidial hydrophobin hyp1 |  | 6 | 0.02 | 3 | 156 | 15.8 | 4.36 | Yes | carbohydrate binding |  |
| maker-contig00040-exonerate_protein2genome-gene-1.33-mRNA-1 | Carbohydrate-binding module family 52 protein |  | 6 | 0.02 | 1 | 745 | 81.3 | 4.86 | Yes | carbohydrate binding |  |
| maker-contig00037-exonerate_protein2genome-gene-1.49-mRNA-1 | unsaturated rhamnogalacturonyl hydrolase | GH105 | 6 | 0.02 | 2 | 157 | 17.4 | 5.19 | yes | carbohydrate metabolic process |  |
| maker-contig00043-exonerate_protein2genome-gene-0.9-mRNA-1 | class iii chitinase i | GH18 | 6 | 0.02 | 1 | 887 | 87 | 4.58 | yes | carbohydrate metabolic process |  |
| maker-contig00004-exonerate_protein2genome-gene-0.80-mRNA-1 | class iii chitinase ii | GH18 | 6 | 0.02 | 1 | 327 | 34.3 | 4.35 | yes | carbohydrate metabolic process |  |
| maker-contig00005-exonerate_protein2genome-gene-6.197-mRNA-1_1 | Glycoside hydrolase family 93 protein | GH93 | 6 | 0.02 | 1 | 400 | 43 | 5.39 | Yes | carbohydrate metabolic process |  |
| maker-contig00219-exonerate_protein2genome-gene-0.0-mRNA-1 | pectate lyase a | PL1 | 6 | 0.02 | 3 | 318 | 33.3 | 6.27 | yes | carbohydrate metabolic process |  |
| maker-contig00009-exonerate_protein2genome-gene-4.247-mRNA-1 | 1-phosphatidylinositol phosphodiesterase |  | 6 | 0.02 | 1 | 382 | 42.7 | 4.88 | yes | Lipid metabolism |  |
| maker-contig00002-exonerate_protein2genome-gene-1.41-mRNA-1 | phosphoglycerate mutase family |  | 6 | 0.02 | 1 | 174 | 19 | 4.27 | Yes | Other functions |  |
| maker-contig00014-exonerate_protein2genome-gene-0.5-mRNA-1_1 | Endonuclease |  | 5 | 0.02 | 3 | 324 | 35.3 | 8.81 | No | Other functions |  |
| maker-contig00037-exonerate_protein2genome-gene-1.15-mRNA-1 | dutp diphosphatase |  | 5 | 0.02 | 3 | 207 | 21.5 | 5.68 | NO | Other functions |  |
| maker-contig00012-exonerate_protein2genome-gene-0.44-mRNA-1 | molecular chaperone hsp70 |  | 5 | 0.02 | 2 | 636 | 69.4 | 5.15 | NO | Other functions |  |
| maker-contig00079-exonerate_protein2genome-gene-0.78-mRNA-1_1 | Glutathione S-transferase GstA |  | 5 | 0.02 | 1 | 252 | 28.9 | 7.03 | NO | Other functions |  |
| maker-contig00044-exonerate_protein2genome-gene-0.105-mRNA-1_2 | Uncharacterized protein |  | 5 | 0.02 | 1 | 425 | 47.1 | 11.74 | NO | Other functions |  |
| maker-contig00018-exonerate_protein2genome-gene-1.29-mRNA-1 | endo-α-mannosidase | GH76 | 5 | 0.02 | 1 | 376 | 40.1 | 4.44 | yes | carbohydrate metabolic process |  |
| maker-contig00016-exonerate_protein2genome-gene-2.0-mRNA-1 | phosphoesterase superfamily protein |  | 5 | 0.02 | 2 | 406 | 45.3 | 4.48 | yes | Other functions |  |
| maker-contig00044-exonerate_protein2genome-gene-1.10-mRNA-1 | 4-aminobutyrate transaminase |  | 4 | 0.02 | 2 | 494 | 54.5 | 8.07 | NO | amino acid metabolism |  |
| P02755 | Beta-lactoglobulin |  | 4 | 0.02 | 2 | 180 | 20 | 5.02 | NO | Other functions |  |
| maker-contig00029-exonerate_protein2genome-gene-0.25-mRNA-1 | polyadenylate-binding protein |  | 4 | 0.02 | 1 | 424 | 44.9 | 5.77 | NO | Other functions |  |
| maker-contig00073-exonerate_protein2genome-gene-0.77-mRNA-1_1 | Serine carboxypeptidase |  | 4 | 0.02 | 1 | 520 | 57.5 | 4.7 | Yes | Amino acid metabolism |  |
| maker-contig00005-exonerate_protein2genome-gene-1.98-mRNA-1 | Endo-1,4-beta-mannanase Man5A | GH5 | 4 | 0.02 | 2 | 299 | 32.2 | 4.53 | yes | carbohydrate metabolic process |  |
| maker-contig00017-exonerate_protein2genome-gene-3.70-mRNA-1_1 | Acyl-CoA dehydrogenase |  | 4 | 0.02 | 2 | 448 | 49.6 | 8.75 | Yes | Oxidases with other functions |  |
| maker-contig00074-exonerate_protein2genome-gene-0.32-mRNA-1 | dipeptidyl 3 |  | 3 | 0.01 | 2 | 774 | 85.9 | 5.67 | NO | Amino acid metabolism |  |
| maker-contig00107-exonerate_protein2genome-gene-0.11-mRNA-1_1 | Aspartyl aminopeptidase |  | 3 | 0.01 | 1 | 530 | 57.8 | 7.18 | NO | amino acid metabolism |  |
| maker-contig00049-exonerate_protein2genome-gene-1.134-mRNA-1 | glucose-6-phosphate isomerase |  | 3 | 0.01 | 2 | 925 | 100.9 | 6.58 | NO | Other functions |  |
| maker-contig00043-exonerate_protein2genome-gene-1.190-mRNA-1 | CipC-like antibiotic response protein |  | 3 | 0.01 | 1 | 119 | 13.6 | 5.48 | No | Other functions |  |
| maker-contig00047-exonerate_protein2genome-gene-1.8-mRNA-1_1 | Aldehyde dehydrogenase |  | 3 | 0.01 | 2 | 498 | 54.4 | 6.34 | NO | Oxidases with other functions |  |
| maker-contig00011-exonerate_protein2genome-gene-0.23-mRNA-1 | calnexin precursor |  | 3 | 0.01 | 1 | 563 | 61.7 | 4.88 | yes | carbohydrate binding |  |
| maker-contig00001-exonerate_protein2genome-gene-9.7-mRNA-1 | cell wall glycosyl hydrolase | GH105 | 3 | 0.01 | 1 | 394 | 44.1 | 5.03 | yes | carbohydrate metabolic process |  |
| maker-contig00041-exonerate_protein2genome-gene-1.36-mRNA-1 | class iii chitinase | GH18-CBM19 | 3 | 0.01 | 1 | 381 | 39.6 | 4.37 | yes | carbohydrate metabolic process |  |
| maker-contig00153-exonerate_protein2genome-gene-0.22-mRNA-1 | alpha-glucuronidase precursor | GH67 | 3 | 0.01 | 1 | 837 | 92 | 5.25 | yes | carbohydrate metabolic process |  |
| Q6BMK0 | Glyceraldehyde-3-phosphate dehydrogenase |  | 3 | 0.01 | 3 | 335 | 35.8 | 6.71 | YES | Other functions |  |
| maker-contig00088-exonerate_protein2genome-gene-0.61-mRNA-1 | mitochondrial hsp70 chaperone |  | 3 | 0.01 | 2 | 671 | 73 | 6.05 | yes | Other functions |  |
| maker-contig00091-exonerate_protein2genome-gene-0.48-mRNA-1_1 | Aspartic-type endopeptidase |  | 2 | 0.01 | 1 | 488 | 51.7 | 5 | NO | Amino acid metabolism |  |
| maker-contig00015-exonerate_protein2genome-gene-4.92-mRNA-1 | lactoylglutathione lyase |  | 2 | 0.01 | 1 | 353 | 40.5 | 6.61 | NO | Other functions |  |
| maker-contig00007-exonerate_protein2genome-gene-2.1-mRNA-1_5 | Phage portal protein |  | 2 | 0.01 | 1 | 1672 | 193.5 | 8.88 | NO | Other functions |  |
| maker-contig00030-exonerate_protein2genome-gene-1.12-mRNA-1 | glycine-rich rna-binding |  | 2 | 0.01 | 1 | 136 | 14.3 | 6.8 | NO | Other functions |  |
| P25272 | Kunitz-type trypsin inhibitor |  | 2 | 0.01 | 1 | 203 | 22.5 | 5.12 | NO | Other functions |  |
| Q1DXH0 | Polyadenylate-binding protein |  | 2 | 0.01 | 1 | 768 | 82.9 | 6.21 | NO | other functions |  |
| maker-contig00011-exonerate_protein2genome-gene-0.80-mRNA-1 | thioredoxin reductase trr1 |  | 2 | 0.01 | 2 | 374 | 39.9 | 5.24 | No | Oxidases with other functions |  |
| P04776 | Glycinin |  | 2 | 0.01 | 1 | 495 | 55.7 | 6.23 | YES | carbohydrate binding |  |
| maker-contig00017-exonerate_protein2genome-gene-2.58-mRNA-1_1 | Beta-mannosidase | GH20 | 2 | 0.01 | 1 | 926 | 103 | 5.02 | Yes | carbohydrate metabolic process |  |
| maker-contig00129-exonerate_protein2genome-gene-0.4-mRNA-1 | beta-1,6 -glucanase | GH30 | 2 | 0.01 | 2 | 480 | 51 | 4.39 | yes | carbohydrate metabolic process |  |
| maker-contig00012-exonerate_protein2genome-gene-3.114-mRNA-1 | pi-plc x domain-containing protein |  | 2 | 0.01 | 1 | 369 | 39.3 | 4.53 | Yes | Lipid metabolism |  |
| maker-contig00045-exonerate_protein2genome-gene-1.131-mRNA-1_1 | ABC-2 type transporter |  | 2 | 0.01 | 1 | 1062 | 117.3 | 5.88 | Yes | Other functions |  |
| maker-contig00081-exonerate_protein2genome-gene-0.10-mRNA-1 | sulfite reductase beta subunit |  | 1 | 0 | 1 | 1525 | 168.2 | 6.06 | NO | Amino acid metabolism |  |
| maker-contig00052-exonerate_protein2genome-gene-0.11-mRNA-1_3 | S-adenosylmethionine synthase |  | 1 | 0 | 1 | 418 | 45.8 | 6.27 | NO | Amino acid metabolism |  |
| maker-contig00009-exonerate_protein2genome-gene-3.66-mRNA-1 | Heat shock chaperonin-binding |  | 1 | 0 | 1 | 565 | 62.5 | 5.5 | NO | Other functions |  |
| maker-contig00002-exonerate_protein2genome-gene-1.29-mRNA-1_2 | Glycyl-tRNA synthetase |  | 1 | 0 | 1 | 639 | 74.7 | 11.82 | NO | Other functions |  |
| maker-contig00008-exonerate_protein2genome-gene-4.0-mRNA-1_1 | NIPSNAP family protein |  | 1 | 0 | 1 | 327 | 38.2 | 8.72 | NO | Other functions |  |
| maker-contig00080-exonerate_protein2genome-gene-0.89-mRNA-1 | pisatin demethylase |  | 1 | 0 | 1 | 345 | 39.3 | 6.34 | NO | Other functions |  |
| maker-contig00031-exonerate_protein2genome-gene-0.21-mRNA-1_5 | Multidrug resistance protein |  | 1 | 0 | 1 | 847 | 96.2 | 8.95 | NO | Other functions |  |
| maker-contig00001-exonerate_protein2genome-gene-11.206-mRNA-1_1 | HypA-like protein |  | 1 | 0 | 1 | 469 | 53.5 | 7.09 | NO | Oxidases with other functions |  |
| maker-contig00015-exonerate_protein2genome-gene-0.0-mRNA-1 | thioredoxin, putative |  | 1 | 0 | 1 | 136 | 14.8 | 8.88 | NO | Oxidases with other functions |  |
| maker-contig00023-exonerate_protein2genome-gene-2.62-mRNA-1_1 | Oxidoreductase molybdopterin binding domain-containing protein |  | 1 | 0 | 1 | 398 | 44.5 | 7.44 | NO | Oxidases with other functions |  |
| maker-contig00064-exonerate_protein2genome-gene-0.21-mRNA-1 | aromatic ring-opening dioxygenase |  | 1 | 0 | 1 | 319 | 35.6 | 7.17 | NO | Oxidases with other functions |  |
| maker-contig00025-exonerate_protein2genome-gene-2.66-mRNA-1_6 | Cytochrome c biogenesis protein ResB |  | 1 | 0 | 1 | 308 | 32.9 | 9.72 | NO | Oxidases with other functions |  |
| maker-contig00079-exonerate_protein2genome-gene-0.9-mRNA-1_1 | Glutaminase |  | 1 | 0 | 1 | 809 | 90.4 | 5.16 | Yes | Amino acid metabolism |  |
| maker-contig00016-exonerate_protein2genome-gene-3.190-mRNA-1 | beta-glucosidase | GH1 | 1 | 0 | 1 | 480 | 54.6 | 5.38 | yes | carbohydrate metabolic process |  |
| maker-contig00110-exonerate_protein2genome-gene-0.35-mRNA-1 | extracellular Polygalacturonase E | GH28 | 1 | 0 | 1 | 410 | 42.5 | 4.77 | yes | carbohydrate metabolic process |  |
| P51640 | Glyceraldehyde-3-phosphate dehydrogenase |  | 1 | 0 | 1 | 312 | 33.5 | 8.85 | Yes | Other functions |  |
|  |  |  |  |  |  |  |  |  |  |  |  |

**Table S2: The distribution of upregulated proteins across the different secretomes containing sugarcane bagasse**

| Names | Total | Elements |  |
| --- | --- | --- | --- |
| 5 g/L Rw  5 g/L PT  10 g/L PT  15 g/L PT | 2 | maker-contig00024-exonerate_protein2genome-gene-0.17-mRNA-1; maker-contig00082-exonerate_protein2genome-gene-0.106-mRNA-1 | beta-galactosidase (GH35); cutinase (CE5) |
| 5 g/L PT  10 g/L PT  15 g/L PT  45 g/L PT | 1 | maker-contig00151-exonerate_protein2genome-gene-0.2-mRNA-1_1 | Carboxylic ester hydrolase |
| 5 g/L PT  10 g/L PT  15 g/L PT | 4 | maker-contig00002-exonerate_protein2genome-gene-4.51-mRNA-1; maker-contig00018-exonerate_protein2genome-gene-3.53-mRNA-1_1; maker-contig00073-exonerate_protein2genome-gene-0.77-mRNA-1_1; maker-contig00008-exonerate_protein2genome-gene-5.150-mRNA-1 | Glucosylceramidase (GH30);  Purple acid phosphatase;  Serine carboxypeptidase;  α-glucosidase agn1 (GH71) |
| 5 g/L PT  10 g/L PT  45 g/L PT | 1 | maker-contig00036-exonerate_protein2genome-gene-2.103-mRNA-1_1 | SMC hinge domain-containing protein |
| 5 g/L PT  15 g/L PT  45 g/L PT | 1 | maker-contig00010-exonerate_protein2genome-gene-2.36-mRNA-1 | Fad-dependent oxidase (AA7) |
| 5 g/L PT  25 g/L PT  45 g/L PT | 1 | maker-contig00011-exonerate_protein2genome-gene-4.30-mRNA-1 | Endo-1,4-beta-xylanase (GH11) |
| 10 g/L PT  15 g/L PT  45 g/L PT | 2 | maker-contig00059-exonerate_protein2genome-gene-1.55-mRNA-1; maker-contig00137-exonerate_protein2genome-gene-0.7-mRNA-1 | Dextranase (GH49); beta-xylosidase (GH3) |
| 5 g/L raw  15 g/L PT | 4 | maker-contig00104-exonerate_protein2genome-gene-0.20-mRNA-1; maker-contig00070-exonerate_protein2genome-gene-0.119-mRNA-1_1; maker-contig00017-exonerate_protein2genome-gene-2.5-mRNA-1; maker-contig00001-exonerate_protein2genome-gene-7.110-mRNA-1 | endo-β-1,4 -xylanase (GH30-CBM1), GMC oxidoreductase; sphingomyelin phosphodiesterase b; hypothetical protein TCE0_018f05417 |
| 5 g/L PT  10 g/L PT | 7 | maker-contig00038-exonerate_protein2genome-gene-2.76-mRNA-1_1; P17493; maker-contig00008-exonerate_protein2genome-gene-3.1-mRNA-1; maker-contig00062-exonerate_est2genome-gene-0.2-mRNA-1_1; P0DO16; maker-contig00015-exonerate_protein2genome-gene-4.29-mRNA-1 P02858 | Carboxypeptidase; Bleomycin resistance protein; histidine phosphatase; Alpha-L-rhamnosidase; Beta-conglycinin; hsp70 chaperone; Glycinin |
| 5 g/L PT  15 g/L PT | 3 | maker-contig00063-exonerate_protein2genome-gene-0.50-mRNA-1; maker-contig00067-exonerate_protein2genome-gene-0.93-mRNA-1; maker-contig00001-exonerate_protein2genome-gene-1.31-mRNA-1_3 | fumarylacetoacetate hydrolase; lactoylglutathione lyase; Transaldolase |
| 10 g/L PT  15 g/L PT | 10 | maker-contig00003-exonerate_protein2genome-gene-5.3-mRNA-1; maker-contig00002-exonerate_protein2genome-gene-1.41-mRNA-1; maker-contig00040-exonerate_protein2genome-gene-1.33-mRNA-1; maker-contig00025-exonerate_protein2genome-gene-1.64-mRNA-1; maker-contig00194-exonerate_protein2genome-gene-0.12-mRNA-1_1; maker-contig00018-exonerate_protein2genome-gene-2.30-mRNA-1; maker-contig00019-exonerate_protein2genome-gene-0.23-mRNA-1_1; maker-contig00007-exonerate_protein2genome-gene-2.1-mRNA-1_5 maker-contig00021-exonerate_protein2genome-gene-2.102-mRNA-1 maker-contig00034-exonerate_protein2genome-gene-0.23-mRNA-1 | Putative β-xylosidase (GH43); phosphoglycerate mutase family; leucine--trna ligase; autophagic serine protease; Uncharacterized protein; suppressor of lurcher protein 1;  Galactan 1,3-beta-galactosidase (GH43); hydrophobic surface binding protein (HsbA); [GPI anchored endo-1,3(4)-beta-glucanase](https://blast.ncbi.nlm.nih.gov/Blast.cgi#alnHdr_GAM41795) (GH16) |
| 25 g/L PT  45 g/L PT | 1 | maker-contig00044-exonerate_protein2genome-gene-0.105-mRNA-1_2 | Hypothetical protein |
| 5 g/L raw | 17 | maker-contig00078-exonerate_protein2genome-gene-0.30-mRNA-1_1; maker-contig00003-exonerate_protein2genome-gene-3.22-mRNA-1_2; maker-contig00035-exonerate_protein2genome-gene-1.82-mRNA-1; maker-contig00011-exonerate_protein2genome-gene-3.42-mRNA-1;  maker-contig00027-exonerate_protein2genome-gene-2.122-mRNA-1_1; maker-contig00013-exonerate_protein2genome-gene-1.72-mRNA-1;  maker-contig00003-exonerate_protein2genome-gene-2.17-mRNA-1; maker-contig00030-exonerate_protein2genome-gene-2.12-mRNA-1; maker-contig00022-exonerate_protein2genome-gene-0.7-mRNA-1_1; maker-contig00014-exonerate_protein2genome-gene-1.10-mRNA-1;  maker-contig00027-exonerate_protein2genome-gene-2.48-mRNA-1_1; maker-contig00066-exonerate_protein2genome-gene-0.3-mRNA-1_1;  maker-contig00001-exonerate_protein2genome-gene-11.72-mRNA-1; maker-contig00018-exonerate_protein2genome-gene-1.29-mRNA-1;  maker-contig00009-exonerate_protein2genome-gene-3.1-mRNA-1_1; maker-contig00006-exonerate_protein2genome-gene-4.70-mRNA-1; maker-contig00001-exonerate_protein2genome-gene-11.206-mRNA-1_1 | Putative glycosyl hydrolase family 16; WD_Repeats domain-containing protein; bifunctional catalase-peroxidase cat2; multidrug transporter;  Dienelactone hydrolase family protein; hypothetical protein TCE0_015r02424; Glycosyl hydrolase (GH2); Protein kinase domain-containing protein; hypothetical protein TCE0_017f04250; Peptidase_S9 domain-containing protein; Endo-1,4-beta-mannosidase (GH5); beta-glucosidase (GH3); endo-α-mannosidase (GH76); Serine hydroxymethyltransferase; aminopeptidase 2; HypA-like protein |
| 5 g/L PT | 7 | maker-contig00105-exonerate_protein2genome-gene-0.59-mRNA-1 Q9UUY8 maker-contig00022-exonerate_protein2genome-gene-2.37-mRNA-1 maker-contig00049-exonerate_protein2genome-gene-1.134-mRNA-1 maker-contig00020-exonerate_protein2genome-gene-3.18-mRNA-1 maker-contig00146-exonerate_protein2genome-gene-0.18-mRNA-1_1 maker-contig00023-exonerate_protein2genome-gene-2.62-mRNA-1_1 | Fad-binding oxidoreductase (AA7); 1,3-beta-glucanosyltransferase (GH72); glucose-6-phosphate isomerase; phosphatidylglycerol specific phospholipase; Mitochondrial glycoprotein; Oxidoreductase molybdopterin binding domain-containing protein |
| 10 g/L PT | 19 | maker-contig00037-exonerate_protein2genome-gene-1.73-mRNA-1; maker-contig00039-exonerate_protein2genome-gene-2.44-mRNA-1; maker-contig00029-exonerate_protein2genome-gene-0.25-mRNA-1; maker-contig00064-exonerate_protein2genome-gene-0.21-mRNA-1; maker-contig00022-exonerate_protein2genome-gene-3.24-mRNA-1; maker-contig00009-exonerate_protein2genome-gene-4.247-mRNA-1; maker-contig00074-exonerate_protein2genome-gene-0.145-mRNA-1; Q02257; maker-contig00035-exonerate_protein2genome-gene-2.65-mRNA-1; maker-contig00074-exonerate_protein2genome-gene-0.108-mRNA-1; maker-contig00025-exonerate_protein2genome-gene-2.66-mRNA-1_6; maker-contig00001-exonerate_protein2genome-gene-11.19-mRNA-1_5; maker-contig00044-exonerate_protein2genome-gene-0.89-mRNA-1_1;  maker-contig00005-exonerate_protein2genome-gene-0.30-mRNA-1_4; maker-contig00043-exonerate_protein2genome-gene-0.0-mRNA-1_5; maker-contig00003-exonerate_protein2genome-gene-4.81-mRNA-1; maker-contig00007-exonerate_protein2genome-gene-4.92-mRNA-1; maker-contig00015-exonerate_protein2genome-gene-4.46-mRNA-1_5 P02755 | Endo- α-mannosidase (GH76); glucan 1,4-alpha-glucosidase (GH31); polyadenylate-binding protein; aromatic ring-opening dioxygenase; aldose 1- epimerase; 1-phosphatidylinositol phosphodiesterase; gpi-anchored cell wall organization protein ecm33; fungistatic metabolite (AA2); allergen asp f7; Cytochrome c biogenesis protein ResB; uncharacterized protein; serine carboxypeptidase; acidPPc domain-containing protein; Uncharacterized protein; IgE-binding protein; endo-beta-glucanase (GH12) |
| 15 g/L PT | 42 | maker-contig00015-exonerate_protein2genome-gene-1.20-mRNA-1; maker-contig00053-exonerate_protein2genome-gene-1.6-mRNA-1; maker-contig00045-exonerate_protein2genome-gene-1.133-mRNA-1; maker-contig00109-exonerate_protein2genome-gene-0.47-mRNA-1_1; maker-contig00009-exonerate_protein2genome-gene-4.228-mRNA-1;  maker-contig00009-exonerate_protein2genome-gene-4.89-mRNA-1; maker-contig00012-exonerate_protein2genome-gene-0.44-mRNA-1; maker-contig00074-exonerate_protein2genome-gene-0.133-mRNA-1_1; maker-contig00014-exonerate_protein2genome-gene-0.47-mRNA-1_1; maker-contig00011-exonerate_protein2genome-gene-1.103-mRNA-1; maker-contig00016-exonerate_protein2genome-gene-2.7-mRNA-1; maker-contig00055-exonerate_protein2genome-gene-0.79-mRNA-1; maker-contig00105-exonerate_protein2genome-gene-0.11-mRNA-1; maker-contig00005-exonerate_protein2genome-gene-2.92-mRNA-1; maker-contig00061-exonerate_protein2genome-gene-1.39-mRNA-1; maker-contig00124-exonerate_protein2genome-gene-0.48-mRNA-1; maker-contig00055-exonerate_protein2genome-gene-1.5-mRNA-1_1; maker-contig00037-exonerate_protein2genome-gene-2.5-mRNA-1; maker-contig00068-exonerate_protein2genome-gene-0.8-mRNA-1; maker-contig00085-exonerate_protein2genome-gene-0.76-mRNA-1; maker-contig00012-exonerate_protein2genome-gene-1.103-mRNA-1; maker-contig00103-exonerate_protein2genome-gene-0.20-mRNA-1; maker-contig00094-exonerate_protein2genome-gene-0.81-mRNA-1; maker-contig00044-exonerate_protein2genome-gene-1.10-mRNA-1; maker-contig00046-exonerate_protein2genome-gene-1.128-mRNA-1; maker-contig00017-exonerate_protein2genome-gene-1.82-mRNA-1; maker-contig00037-exonerate_protein2genome-gene-1.51-mRNA-1; maker-contig00022-exonerate_protein2genome-gene-0.79-mRNA-1; maker-contig00079-exonerate_protein2genome-gene-0.41-mRNA-1; maker-contig00077-exonerate_protein2genome-gene-0.106-mRNA-1_6; maker-contig00087-exonerate_protein2genome-gene-0.17-mRNA-1; maker-contig00080-exonerate_protein2genome-gene-0.55-mRNA-1; maker-contig00030-exonerate_protein2genome-gene-1.12-mRNA-1; maker-contig00079-exonerate_protein2genome-gene-0.78-mRNA-1_1; maker-contig00034-exonerate_protein2genome-gene-2.61-mRNA-1; maker-contig00005-exonerate_protein2genome-gene-2.76-mRNA-1; maker-contig00005-exonerate_protein2genome-gene-4.25-mRNA-1; maker-contig00028-exonerate_protein2genome-gene-2.59-mRNA-1_1; maker-contig00047-exonerate_protein2genome-gene-1.3-mRNA-1; maker-contig00041-exonerate_protein2genome-gene-1.36-mRNA-1; maker-contig00153-exonerate_protein2genome-gene-0.22-mRNA-1; maker-contig00064-exonerate_protein2genome-gene-0.60-mRNA-1_1 | hypothetical protein TCE0_015f01617; genome polyprotein (GH127); isoamyl alcohol oxidase (AA7); Alpha-trehalose glucohydrolase TreA (GH65); glucan endo- -alpha-glucosidase agn1 (GH71); mrna splicing factor rna helicase; molecular chaperone hsp70; alpha-L-arabinofuranosidase (GH51-CBM42); GPI anchored protein; Lipase 1 (CE10); sun domain protein (GH132); Carboxylic ester hydrolase (CE10); proline iminopeptidase; swollenin; bnr asp-box repeat domain protein (GH93); Beta-galactosidase (GH35); Phenazine biosynthesis-like protein; extracellular dihydrogeodin oxidase; hypothetical protein TCE0_011f00638 (GH93); ML domain-containing protein; virulence protein stm3117; Peptide hydrolase; beta-glucosidase (GH3); 4-aminobutyrate transaminase; hypothetical protein TCE0_044r17570; amidase family protein; polysaccharide lyase (PL1); Endo-1,4-beta-xylanase (GH11); ubiquitin-40s ribosomal protein; Sensor histidine kinase; 3-isopropylmalate dehydrogenase; glycine-rich RNA-binding; Glutathione S-transferase GstA; Allantoicase; alpha-l-arabinofuranosidase (GH61); alkaline serine protease; Glucan endo-1,3-alpha-glucosidase (GH71); 1,3-beta-glucanosyltransferase (GH72); chitinase (GH18-CBM19); alpha-glucuronidase precursor (GH67); Beta-xylosidase (GH5) |
| 25 g/L PT | 2 | maker-contig00026-exonerate_protein2genome-gene-2.30-mRNA-1; maker-contig00106-exonerate_protein2genome-gene-0.28-mRNA-1_3 | **Endo-polygalacturonase (GH28);** GPI-anchored cell wall beta-1,3-endoglucanase (GH17) |
| 45 g/L PT | 4 | maker-contig00010-exonerate_protein2genome-gene-1.9-mRNA-1 maker-contig00007-exonerate_protein2genome-gene-3.34-mRNA-1_6 maker-contig00047-exonerate_protein2genome-gene-0.53-mRNA-1 maker-contig00014-exonerate_protein2genome-gene-4.97-mRNA-1_6 | fructose-bisphosphate aldolase;  ATP-binding cassette transporter; acetyl xylan esterase (CE5-CBM1); Biotin carboxylase |

**Rw refers to the raw sugarcane bagasse while PT refer to the pretreated sugarcane bagasse
